# Antimony-ligated dysprosium single-molecule magnets as catalysts for stibine dehydrocoupling†
†Electronic supplementary information (ESI) available: Synthetic details, spectroscopic characterization for all compounds, X-ray crystallography details and crystallographic information files, computational details. CCDC 1484570–1484573 and 1485316. For ESI and crystallographic data in CIF or other electronic format see DOI: 10.1039/c6sc04465d



**DOI:** 10.1039/c6sc04465d

**Published:** 2016-11-21

**Authors:** Thomas Pugh, Nicholas F. Chilton, Richard A. Layfield

**Affiliations:** a School of Chemistry , The University of Manchester , Oxford Road , Manchester , M13 9PL , UK . Email: Richard.Layfield@manchester.ac.uk ; Email: Nicholas.Chilton@manchester.ac.uk

## Abstract

Single-molecule magnets (SMMs) are coordination compounds that exhibit magnetic bistability below a characteristic blocking temperature. Research in this field continues to evolve from its fundamental foundations towards applications of SMMs in information storage and spintronic devices. Synthetic chemistry plays a crucial role in targeting the properties that could ultimately produce SMMs with technological potential. The ligands in SMMs are invariably based on non-metals; we now report a series of dysprosium SMMs (in addition to their magnetically dilute analogues embedded in yttrium matrices) that contain ligands with the metalloid element antimony as the donor atom, *i.e.* [(η^5^-Cp′_2_Dy){μ-Sb(H)Mes}]_3_ (**1-Dy**) and [(η^5^-Cp′_2_Dy)_3_{μ-(SbMes)_3_Sb}] (**2-Dy**), which contain the stibinide ligand [Mes(H)Sb]^–^ and the unusual Zintl-like ligand [Sb_4_Mes_3_]^3–^, respectively (Cp′ = methylcyclopentadienyl; Mes = mesityl). The zero-field anisotropy barriers in **1-Dy** and **2-Dy** are *U*_eff_ = 345 cm^–1^ and 270 cm^–1^, respectively. Stabilization of the antimony-ligated SMMs is contingent upon careful control of reaction time and temperature. With longer reaction times and higher temperatures, the stibine pro-ligands are catalytically dehydrocoupled by the rare-earth precursor complexes. NMR spectroscopic studies of the yttrium-catalysed dehydrocoupling reactions reveal that **1-Y** and **2-Y** are formed during the catalytic cycle. By implication, **1-Dy** and **2-Dy** should also be catalytic intermediates, hence the nature of these complexes as SMMs in the solid-state and as catalysts in solution introduces a strategy whereby new molecular magnets can be identified by intercepting species formed during catalytic reactions.

## Abstract 2

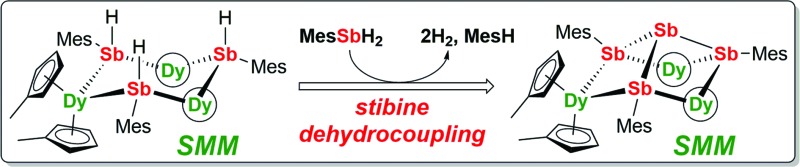
The synthesis of antimony-ligated dysprosium SMMs is described in addition to the unexpected reactivity of the SMMs in stibine dehydrocoupling catalysis.

## Introduction

The synthesis of complex magnetic materials from simple chemical building blocks encapsulates the intrinsic fascination of molecular magnetism. Molecular magnets are typically designed using bottom-up approaches that provide access to experimental testbeds for theoretical models of magnetism, and that enable modular approaches to applications based on the properties of well-defined magnetic units. For example, carefully constructed transition metal complexes display characteristics that could lead to their implementation as molecular qubits for quantum computing.^1^ The magnetocaloric effect, in which the entropy of a magnetic system is modulated by a magnetic field, introduces the possibility of using molecular magnets as refrigerants that function more efficiently than conventional cryogens.^2^ Spin-crossover materials, which have been intensively studied for many years,^3^ have been proposed for applications in displays, sensors and information storage devices.^4^ Lanthanide complexes continue to play important roles in enhancing our understanding of ligand field theory,^5^ and many such species find applications in NMR spectroscopy as shift reagents^6^ and in magnetic resonance imaging.^7^

Single-molecule magnets (SMMs) are coordination compounds that can be defined by an effective energy barrier (*U*_eff_) to reversal of their magnetization.^8^ The pioneering work on SMMs focused on exchange-coupled transition metal cage compounds,^9,10^ and monometallic 3d complexes have recently emerged as another important class of SMM.^11–13^ Many of the most exciting developments in single-molecule magnetism have been accounted for by the lanthanides terbium, dysprosium and erbium,^14–19^ and lanthanide SMMs have been described with very high *U*_eff_ values and magnetic blocking temperatures.^20–22^ Studies of the interactions between electrical currents and SMMs on surfaces has also led to the development of prototype molecular spintronic devices.^23,24^

Despite the remarkable progress with SMMs, challenges remain, including overcoming the need for liquid-helium temperatures to observe slow relaxation of the magnetization, and the need to organise and stabilise molecules on surfaces for devices to become viable. To address these challenges, novel synthetic coordination chemistry strategies are of prime importance. Ligand environments in SMMs, especially those containing lanthanides, are dominated by hard, oxygen- and nitrogen-donor atoms.^8–22^ Targeting lanthanide SMMs with ligands in which the donor atoms have metallic character would introduce new ways of influencing the metal–ligand bonding and hence the electronic structure of the metal ion, potentially providing a way of enhancing the magnetic relaxation properties. Furthermore, using metalloid donor ligands as building blocks in SMMs could unearth new reactivity, which could itself be manipulated further for the synthesis of new molecular magnets.

## Results and discussion

**Scheme 1:**
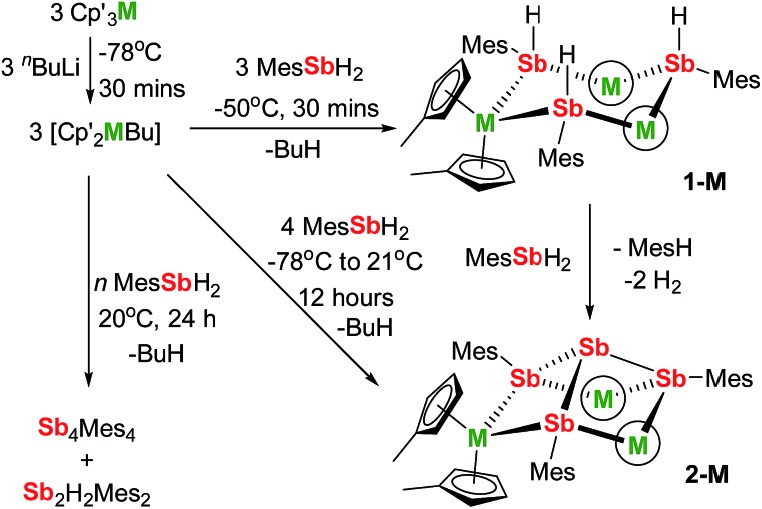
Synthesis of **1-M** and **2-M** (M = Dy, Y; Mes = mesityl; the circled M represents Cp′_2_M).

We now describe two dysprosium-containing SMMs based on the metallocene building block {Cp_2_Dy(E)_2_}, where E denotes a ligand with the 5p metalloid element antimony as the donor atom. We focus on the trimetallic complexes [(η^5^-Cp′_2_Dy){μ-Sb(H)Mes}]_3_ (**1-Dy**) and [(η^5^-Cp′_2_Dy)_3_{μ-(SbMes)_3_Sb}] (**2-Dy**), which contain the stibinide ligand [Mes(H)Sb]^–^ and the Zintl-like ligand [Sb_4_Mes_3_]^3–^, respectively (Cp′ = methylcyclopentadienyl; Mes = mesityl). Compound **1-Dy** and the yttrium analogue **1-Y** were synthesized by adding three equivalents of MesSbH_2_ to a 3 : 3 mixture of Cp′_3_M and ^*n*^BuLi over 30 minutes at –50 °C (Scheme 1). Compounds **2-M** were synthesized in a similar fashion with four equivalents of MesSbH_2_, with the reaction being warmed from –78 °C to room temperature overnight. Without careful control of reaction time and temperature, stibine dehydrocoupling occurs to give the 1,2-distibane Sb_2_H_2_Mes_2_ (Fig. S1†), the tetrastibetane Sb_4_Mes_4_,^25,26^ and H_2_. This unanticipated reactivity introduced the possibility of converting **1-M** into **2-M** *via* cross-dehydrocoupling of the former with MesSbH_2_: in the case of **2-Y**, the reaction is quantitative by ^1^H NMR spectroscopy (Fig. S5†), and for **2-Dy** the isolated yield was 45%. The dehydrocoupling reactivity is considered further after discussion of the structural and magnetic properties of **1-Dy** and **2-Dy**.

**Fig. 1:**
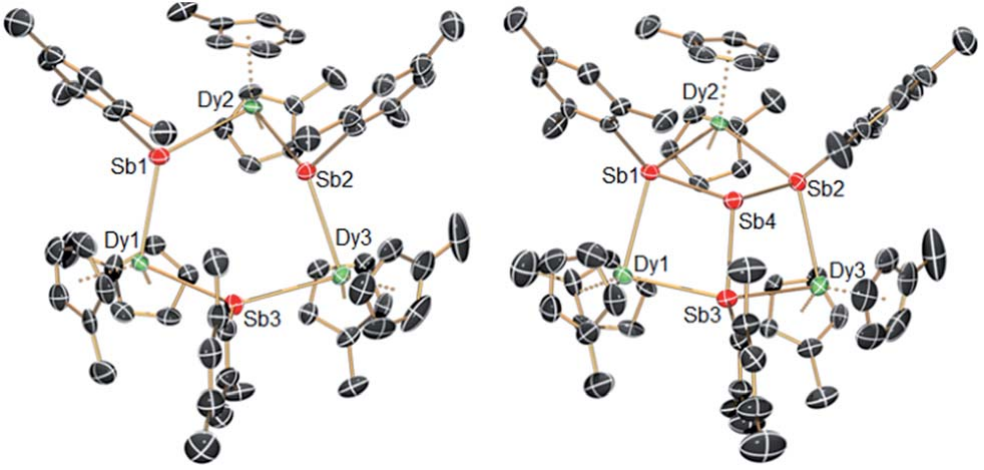
Molecular structures of **1-Dy** (left) and **2-Dy** (right). Thermal ellipsoids at the 50% probability level. Hydrogen atoms not shown.

The structures of **1-M** and **2-M** were determined by X-ray diffraction (Fig. 1, Tables S1 and S2†). Each compound contains a chair-like M_3_Sb_3_ ring in which the metal atoms bond to two μ-[Mes(H)Sb]^–^ ligands in **1-M** or to two antimony atoms of the [Sb_4_Mes_3_]^3–^ ligand in **2-M**. The metal atoms also bond to two η^5^-Cp′ ligands, such that they adopt pseudo-tetrahedral geometries. The central antimony atom in **2-M** connects the three μ-{MesSb} groups, with Sb–Sb distances of 2.8583(11)–2.8687(11) Å in **2-Dy**. The Dy_3_Sb_3_ rings in both dysprosium compounds are similar in size, with the Dy–Sb bond lengths in **1-Dy** being 3.092(6)–3.212(3) Å, and those in **2-Dy** 3.119(1)–3.138(1) Å. The Dy···Dy separations are 5.7174(7)–5.8535(5) Å and 5.7175(8)–5.8293(8) Å in **1-Dy** and **2-Dy**, respectively. The Sb–Dy–Sb angles are 87.53(8)–103.85(12)° in **1-Dy** and 85.32(3)–89.15(3)° in **2-Dy**; the Dy–Sb–Dy angles are 128.26(3)–136.73(14)° and 132.03(3)–136.96(3)°, respectively. The Dy–C distances in **1-Dy** and **2-Dy** are 2.59(1)–2.651(9) Å and 2.58(1)–2.66(1) Å, and the Cp_cent_–Dy–Cp_cent_ angles are 129.30(17)–130.92(19)° and 129.9(2)–130.3(2)°. The geometric parameters for **1-Y** and **2-Y** are similar to those of their dysprosium analogues (Fig. S2 and Table S2†). The *C*_1_ symmetry of **1-Y** and **2-Y** in the solid state is reflected in their ^1^H NMR spectra, which show multiple resonances for the inequivalent CH and CH_3_ groups in both molecules (Fig. S3 and S4†). Characteristic Sb–H stretches were observed in the IR spectra of **1-M** at 1860–1875 cm^–1^ (Fig. S6†).

Molecular rare-earth complexes of antimony ligands are extremely rare. The sole prior example of a Zintl-ligated rare-earth complex is 
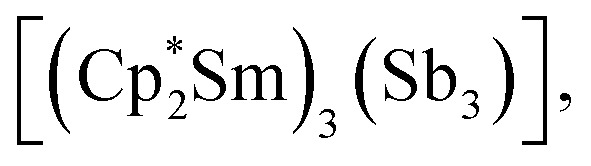
 which contains the chain-like [Sb_3_]^3–^ ligand.^27^ The [Sb_4_R_3_]^3–^ ligand motif is itself extremely rare, with the only previous example being found in [(Cp_2_Ti)_3_{(SbR)_3_Sb}], which forms in the reaction of [Cp_2_Ti{C_2_(SiMe_3_)_2_}] with Sb_2_R_4_ (R = 2-(Me_2_NCH_2_)C_6_H_4_).^28^

### Magnetic properties

The magnetic susceptibilities of **1-Dy** and **2-Dy** were measured in a d.c. field of 1 kOe. The plots of *χ*_M_ *T*(*T*) in the range 2–300 K for both compounds are similar (Fig. S7†) and consistent with the presence of three Dy^3+^ ions with ^6^H_15/2_ ground terms and *g*_*J*_ = 4/3 (theoretical *χ*_M_ *T* = 42.5 cm^3^ K mol^–1^ at 300 K). For **1-Dy**, *χ*_M_ *T* is 40.57 cm^3^ K mol^–1^ at 300 K before gradually decreasing upon cooling to 50 K; at lower temperatures the decrease in *χ*_M_ *T* is more rapid, reaching 10.01 cm^3^ K mol^–1^ at 2 K. The values of *χ*_M_ *T* for **2-Dy** at 300 K and 2 K are 42.69 cm^3^ K mol^–1^ and 10.10 cm^3^ K mol^–1^, respectively. The field (*H*) dependence of the magnetization (*M*) at 1.8 K is also similar for **1-Dy** and **2-Dy**, with both showing a steep increase in *M* as the field increases to 20 kOe, and then a slower increase, before reaching 15.17 *μ*_B_ and 15.82 *μ*_B_, respectively, at 70 kOe (Fig. S8†).

Comparing the experimental and calculated magnetic properties for **1-Dy** and **2-Dy** in the absence of intramolecular exchange interactions, it is clear that the experimental decrease in *χ*_M_ *T* at low temperatures cannot be due to ligand field effects alone. Similarly, the increase in magnetization at low fields is slower than calculated. These observations imply non-negligible antiferromagnetic exchange interactions between the dysprosium centres, which were simulated by implementing the Lines model^29,30^ and the Hamiltonian shown in eqn (1) using PHI.^31^

1

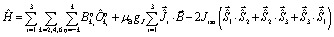

Here, the *Ô* *q* *k_i_* operator equivalents act on the |*J*, *m*_*J*__*i*_ basis of the ^6^H_15/2_ term of each Dy^3+^ ion where the *B* *q* *k_i_* crystal field terms are fixed from CASSCF calculations (see below) taking into account the relative orientations of the local reference frames of each Dy^3+^ ion. The only variable is the single isotropic Lines exchange constant *J*_iso_, which acts on the true *S* = 5/2 spins of the Dy^3+^ ions *via* a Clebsch–Gordan decoupling; we use this term to account for both the exchange and dipolar coupling. Modelling the interactions in this way, the best simulations are obtained for **1-Dy** and **2-Dy** using *J*_iso_ = –0.121 cm^–1^ and –0.150 cm^–1^, respectively (Fig. S7 and S8†).

**Fig. 2:**
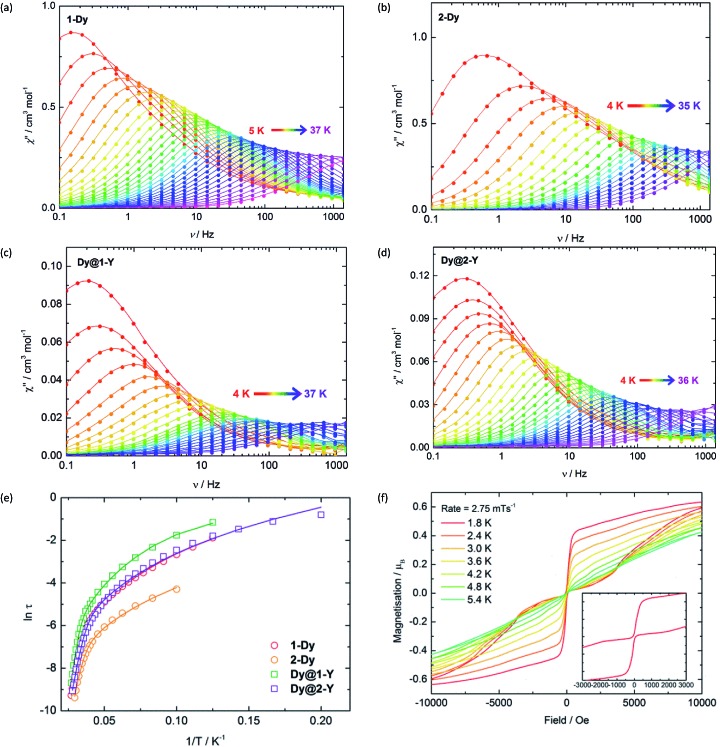
Frequency dependence of the out-of-phase (*χ*′′) magnetic susceptibility for: (a) **1-Dy**; (b) **2-Dy**; (c) **Dy@1-Y**; (d) **Dy@2-Y**. (e) Temperature dependence of the magnetization relaxation times (*τ*) plotted as ln(*τ*/s) *vs. T* ^–1^, with the solid lines representing theoretical fits using the parameters in Table 1. (f) Magnetization (*M*) *vs.* field (*H*) hysteresis loops for **Dy@1-Y** with a scan rate of 2.8 mT s^–1^.

The SMM properties of **1-Dy** and **2-Dy** were investigated using a.c. magnetic susceptibility measurements, employing a weak a.c. field of 1.55 Oe and zero d.c. field. In order to explore the impact of exchange interactions on the SMM properties, we also studied the magnetically dilute analogues [(Cp′_2_Dy)(Cp′_2_Y)_2_{Sb(H)Mes}]_3_ (**Dy@1-Y**) and [(Cp′_2_Dy)(Cp′_2_Y)_2_{(SbMes)_3_Sb] (**Dy@2-Y**). Dilution levels of 5% were achieved by combining Cp′_3_Y and Cp′_3_Dy in 19 : 1 ratio and performing the syntheses according to Scheme 1, which produced **Dy@1-Y** and **Dy@2-Y** in matrices of **1-Y** and **2-Y**, respectively. The frequency (*ν*) dependence of the in-phase (*χ*′) (Fig. S9 and S10†) and the out-of-phase (*χ*′′) (Fig. 2) magnetic susceptibilities reveal prominent SMM behaviour for **1-Dy** and **2-Dy**. The *χ*′′(*ν*) plots for both systems show well-defined maxima in the temperature range 5–36 K and 4–33 K, respectively, using a.c. frequencies up to 1400 Hz. The plots of *χ*′′ *vs. χ*′ for the undiluted SMMs are semi-circular in nature, and were fitted using a modified Debye model with *α* parameters of 0.20–0.52 and 0.19–0.40 for **1-Dy** and **2-Dy**, respectively, indicating broad distributions of relaxation times (Fig. S11†). The diluted systems **Dy@1-Y** and **Dy@2-Y** also show pronounced SMM behaviour, with maxima in *χ*′′(*ν*) being observed up to slightly higher temperatures relative to the undiluted SMMs (Fig. 2, S12 and S13†). The *α* parameters for the dilute SMMs are 0.25–0.44 and 0.03–0.43 for **Dy@1-Y** and **Dy@2-Y**, respectively (Fig. S14†).

Insight into the relaxation dynamics of the SMMs was obtained by plotting ln *τ versus T* ^–1^ (Fig. 2), where *τ* is the relaxation time. The four SMMs display similar properties, where the high-temperature regimes show a linear dependence of ln *τ* on *T* ^–1^, indicating relaxation *via* Orbach and/or thermally assisted quantum tunnelling of the magnetization (TA-QTM) mechanisms. At lower temperatures, the relaxation shows a weaker temperature dependence, suggesting relaxation *via* a Raman process; as the experiment was conducted in zero field, relaxation *via* the direct process is expected to be negligible. Notably, the relaxation dynamics do not enter a temperature-independent regime (usually assigned to ground-state QTM) at the lowest temperatures attainable by our SQUID magnetometer. The data was modelled for each SMM using the equation *τ* ^–1^ = *τ*_0_^–1^e^–*U*_eff_/*k*_B_ *T*^ + *CT* ^*n*^, where *τ*_0_ and *U*_eff_ are the Orbach parameters, and *C* and *n* are the Raman parameters (Table 1). The *U*_eff_ value of 345 cm^–1^ for **1-Dy** is one of the largest yet determined for a polymetallic SMM in zero applied field. The highest anisotropy barriers in SMMs based on lanthanide ions with oblate electron density in the most magnetic *m*_*J*_ states – such as Dy^3+^ – typically occur when strong crystal fields are applied on high-order symmetry axes.^8^ Thus, the current record anisotropy barrier is 1261 cm^–1^, which was determined for a *D*_5h_-symmetric dysprosium complex with a pentagonal bipyramidal arrangement of donor atoms.^21^ In light of this, a remarkable observation on **1-Dy** is that a very large barrier can still be obtained when the Dy^3+^ occupies a much lower symmetry environment of approximately *C*_2v_ (assuming ring whizzing of the Cp′ ligands). The Raman exponents *n* are similar to those in other metallocene-based SMMs.^32^

**Table 1:** Parameters used to fit the magnetization dynamics of **1-Dy**, **Dy@1-Y**, **2-Dy** and **Dy@2-Y**

	**1-Dy**	**Dy@1-Y**	**2-Dy**	**Dy@2-Y**
*U*_eff_/cm^–1^	345	345	272	270
*τ*_0_/s	1.57 × 10^–10^	2.96 × 10^–10^	1.10 × 10^–9^	2.87 × 10^–9^
*C*/s^–1^ K^–*n*^	6.15 × 10^–3^	2.57 × 10^–3^	0.128	6.36 × 10^–3^
*n*	3.35	3.39	2.72	3.30

Variable-field magnetization measurements on the SMMs revealed marked differences between the non-dilute and dilute systems. For **1-Dy**, a sweep rate of 2 mT s^–1^ produced a narrow S-shaped hysteresis loop at 1.8 K (Fig. S15†), whereas butterfly-shaped loops were observed for **Dy@1-Y** at 1.8–5.4 K (Fig. 2). The hysteresis properties of **2-Dy** and **Dy@2-Y** (Fig. 2 and S15†) mirror those of the stibine-ligated compounds, albeit with the *M*(*H*) loops for the diluted system remaining open up to 4.0 K. The likeliest explanation for the closed hysteresis loops in the non-dilute SMMs is that exchange interactions between the Dy^3+^ ions provide tunnelling pathways that close upon replacement with diamagnetic Y^3+^. The precipitous drop in magnetization for the diluted SMMs around zero field is characteristic of the vast majority of SMMs and can be attributed to single-ion effects such as hyperfine coupling to spin-active isotopes of dysprosium.^33^

### Theoretical characterization

**Fig. 3:**
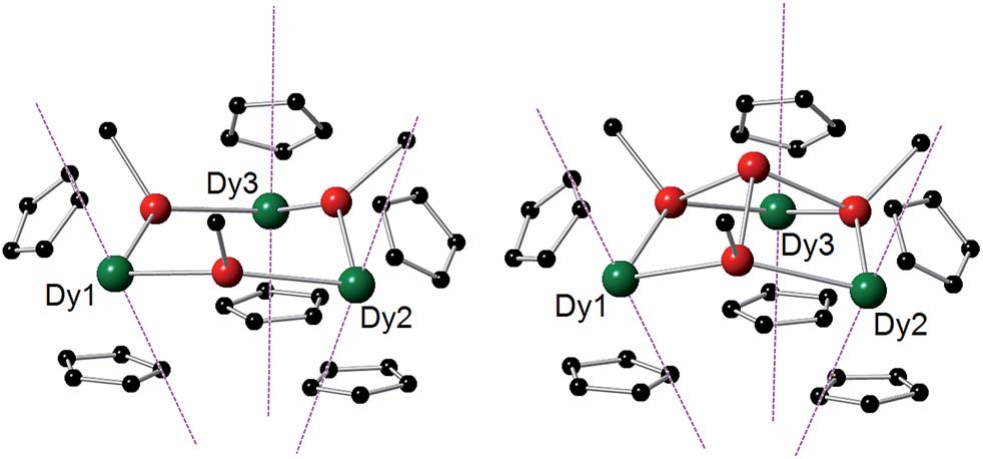
Orientations of the main magnetic axes for the ground doublets of the Dy^3+^ ions in **1-Dy** (left) and **2-Dy** (right). The magnetic axes are shown as dashed purple lines. Dy = green, Sb = red, C = black. For clarity, only the *ipso* carbons of the mesityl substituents are shown, and the Cp′ methyl groups and hydrogen atoms have been omitted.

Deeper insight into the magnetic properties of **1-Dy** and **2-Dy** was obtained by performing complete active space self-consistent field (CASSCF) calculations.^34^ For both complexes, the electronic structure of the individual Dy^3+^ ions is dominated by the [Cp′]^–^ ligands, which creates a strong axial potential and leads to the ground Kramers doublet at each Dy^3+^ ion being well described as *m*_*J*_ = ±15/2. The main magnetic anisotropy axis of each Dy^3+^ ion is therefore oriented along the local [Cp′]···[Cp′] direction, where all three form a teepee-like arrangement (Fig. 3). The dominant axial potential generated by the [Cp′]^–^ ligands also results in the first- and second-excited states being highly axial in nature and collinear with the ground-state axis for all sites in **1-Dy** and **2-Dy** (Tables 2 and S3–S8†).

**Table 2:** Properties of the four lowest-energy Kramers doublets in **1-Dy** and **2-Dy**. Energies and *g*-tensor values are averaged across the three Dy^3+^ sites in each molecule. Standard deviations are given in brackets. ‘Angle’ refers to the orientation of the magnetic axis relative to that in the ground doublet

Doublet	Energy/cm^–1^	*g*_*x*_	*g*_*y*_	*g*_*z*_	Angle/°
**1-Dy**					
1	0	0.00	0.00	19.57(5)	
2	167(3)	0.00	0.00	17.04(7)	2.6(7)
3	329(5)	0.04(3)	0.05(3)	14.74(3)	3.5(9)
4	416(3)	2(1)	4(3)	11(1)	24(33)

**2-Dy**					
1	0	0.00	0.00	19.66(1)	
2	166(1)	0.00	0.00	17.12(3)	3.0(5)
3	324(9)	0.03(2)	0.04(3)	14.69(5)	1.6(5)
4	417(13)	0.6(3)	0.7(3)	11.6(2)	3.9(7)

The *C*_2v_ symmetry of the dysprosium environments renders a rhombic third excited state in both complexes; this is likely to be the origin of the most efficient thermal relaxation pathway in **1-Dy** since the rhombic state is calculated to lie at 416(3) cm^–1^, which is comparable to the experimental *U*_eff_ value of 345 cm^–1^. For **2-Dy**, the rhombic third excited state lies at 413(17) cm^–1^, which is much larger than the experimental barrier of 270 cm^–1^. Although relaxation *via* higher-lying Kramers' doublets is known,^35–37^ it remains a relatively uncommon phenomenon, with thermally activated relaxation thought to proceed *via* the first-excited doublet in most SMMs.^38^ In both cases, magnetic dilution does not significantly alter the a.c. susceptibility properties, hence the discrepancy between theory and experiment for **2-Dy** cannot arise from intramolecular interactions. Despite the differing ligand environments in **1-Dy** and **2-Dy**, the properties of the low-lying Kramers doublets in both complexes are remarkably similar, as are the orientations of the ground-state anisotropy axes. The LoProp charges on the antimony atoms bonded to the Dy^3+^ centres range from –0.17 to –0.23 for **1-Dy** and from –0.28 to –0.29 for **2-Dy**, respectively.^39^ Although the accumulation of charge on the donor atoms is not large in either case, the negligible difference in the average Dy–Sb bond lengths of 0.036 Å between the two systems combined with the slightly greater charge density on the antimony atoms in the equatorial plane in **2-Dy** relative to **1-Dy** can account for the lower *U*_eff_ value in the former, which is consistent with observations on related SMMs containing [MesE(H)]^–^ and [MesE]^2–^ ligands (E = P, As).^40,41^ The *U*_eff_ value determined for **1-Dy** of 345 cm^–1^ is markedly larger than those determined for the isostructural phosphide- and arsenide-bridged analogues [(η^5^-Cp′_2_Dy){μ-E(H)Mes}]_3_ (E = P, As), of 210 cm^–1^ and 256 cm^–1^, respectively. The only significant differences in the molecular structures of **1-Dy** and the two lighter congeners are the dysprosium–pnictogen bond lengths, which increase significantly with the radius of pnictogen (those in **1-Dy** are, on average, 0.168 Å longer than those in the As-bridged analogue). Since the main magnetic axes in the phosphide-, arsenide- and stibinide-bridged SMMs all adopt similar orientations along the [Cp′]···[Cp′] directions, the pnictogens occupy equatorial sites; as the Dy–E bond lengths increase, the influence of the pnictogen on the splitting of the Dy^3+^ crystal field levels diminishes, leading to a more dominant axial crystal field and hence larger *U*_eff_ values.

Being intrigued by the unusual [Sb_4_Mes_4_]^3–^ ligand, we endeavoured to determine the electronic structure of this species. The Dy^3+^ ions in **2-Dy** were replaced with Lu^3+^ to ensure a well-defined active space for the antimony-containing ligand, and the restricted active space (RAS) probing approach was employed with **2-Lu** to identify an appropriate orbital manifold near the Fermi level to describe the Sb_4_ unit. The resulting CAS, which consisted of 12 electrons in 9 orbitals for the lowest lying ten *S* = 0 and ten *S* = 1 states delocalized over the Sb_4_ unit, is dominated by the antimony 5p orbitals (Fig. S16†). The ground state of [Sb_4_Mes_4_]^3–^ is a well-isolated *S* = 0, as expected, however after the first excitation to the *S* = 1 state at *ca.* 26 000 cm^–1^, there is a continuum of states up to at least 45 000 cm^–1^ (Table S9 and Fig. S17†). This delocalized set of continuum states is reminiscent of a semi-conductor, and it is possible that this feature also contributes to diminishing *U*_eff_ in **2-Dy**. Unfortunately, however, all efforts to calculate the properties of the individual Dy^3+^ ions while allowing excitation into the Sb_4_ continuum failed owing to the extremely large active space required for the calculation.

### Stibine dehydrocoupling reactivity

**Fig. 4:**
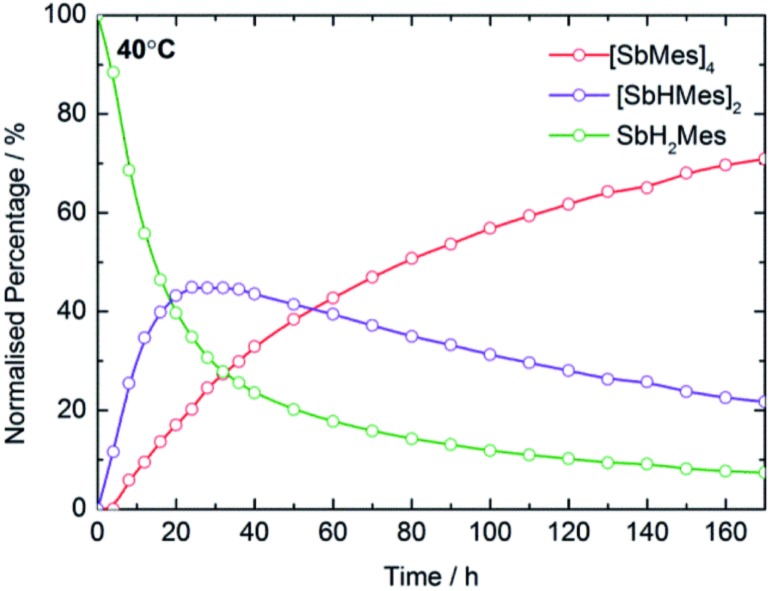
Product distribution as a function of time at 40 °C for the dehydrocoupling of MesSbH_2_ initiated by 10 mol% Cp′_3_Y.

In optimizing the synthesis of **1-M** and **2-M**, it was apparent from ^1^H NMR spectroscopic studies of the yttrium derivatives that **1-Y**, **2-Y**, Sb_2_H_2_Mes_2_, Sb_4_Mes_4_ and H_2_ all form during the same reaction. Furthermore, the relative amounts of each component depend on reaction time and temperature, with longer times and higher temperatures producing greater amounts of Sb_4_Mes_4_. These observations implied that yttrium mediates – or even catalyses – the dehydrocoupling of MesSbH_2_. To investigate this possibility, ^1^H NMR spectroscopy was used to study the reactions of MesSbH_2_ with 10 mol% of Cp′_3_Y at 40, 50, 60 and 70 °C (Fig. S18–S21†). The initial ^1^H NMR spectrum of the 40 °C reaction shows the resonances of the two starting materials (Fig. S18†), whereas after 20 hours MesSbH_2_, Sb_2_H_2_Mes_2_ (both diastereomers) and Sb_4_Mes_4_, account for 40%, 43% and 17% of the antimony-containing species (Fig. 4). The ^1^H NMR spectrum also shows H_2_ at 4.47 ppm. The amount of Sb_2_H_2_Mes_2_ then gradually decreases, accounting for 20% after 170 h, whereas the amount of Sb_4_Mes_4_ increases to reach 71%, with 7% MesSbH_2_ remaining. Also noteworthy in the ^1^H NMR spectrum is the rapid emergence of a series of broad resonances in the region *δ* ≈ 6.1–6.6 ppm, which correspond to the methine CH resonances of **1-Y**. After 170 h, all the signals due to **1-Y** have been replaced by those of **2-Y**. At 50 °C, the conversion of MesSbH_2_ to Mes_4_Sb_4_ increases to 80% at a faster rate, but at higher temperatures the conversion level decreases and, at 70 °C, an appreciable amount of mesitylene was observed due to decomposition of MesSbH_2_ (Fig. S22†). Thus, Cp′_3_Y does catalyse the dehydrocoupling of MesSbH_2_ to give Sb_2_H_2_Mes_2_ and then Sb_4_Mes_4_.

The initial yttrium-containing product of the dehydrocoupling is **1-Y**, which is subsequently converted into **2-Y**. Since our stoichiometric (Scheme 1) and catalytic reaction studies have established that **1-Y** reacts quantitatively with MesSbH_2_ to give **2-Y** (Fig. S5†), the fate of **2-Y** once formed is of interest. This was probed by adding 3.33 mol% of **2-Y** (*i.e.* 10 mol% yttrium) to MesSbH_2_ and following the reaction by ^1^H NMR spectroscopy at 40 °C (Fig. S23†). The resulting spectra acquired over 345 h reveal that, although the reaction is slower than with Cp′_3_Y as the catalyst, **2-Y** does dehydrocouple MesSbH_2_ to give Sb_2_H_2_Mes_2_ and H_2_, and then Sb_4_Mes_4_.

**Scheme 2:**
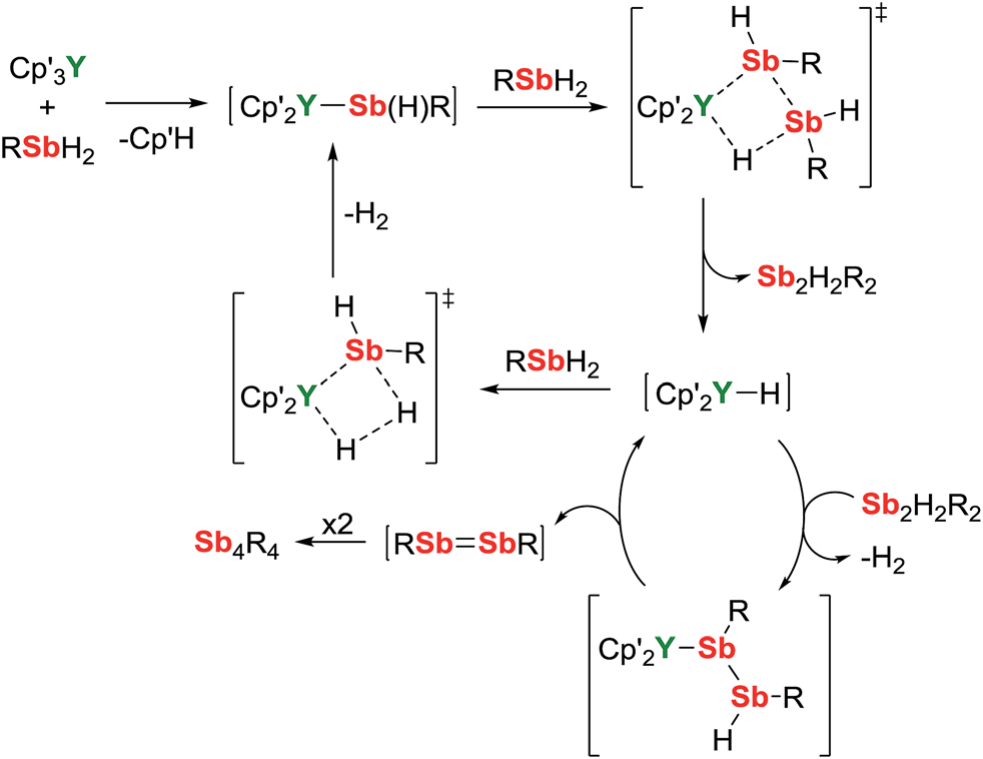
Dehydrocoupling of MesSbH_2_ catalysed by Cp′_3_Y (R = mesityl).

A mechanism for the catalytic dehydrocoupling of MesSbH_2_ by Cp′_3_Y is proposed in Scheme 2. The variation in the relative amounts of MesSbH_2_, Sb_2_H_2_Mes_2_ and Sb_4_Mes_4_ as a function of time, in addition to the formation of H_2_, suggests: (i) that the distibine is formed from dehydrocoupling of MesSbH_2_, and; (ii) that the tetrastibetane is formed subsequently from further reactivity of the distibine. The formation of Sb_2_H_2_Mes_2_ also implies that the dehydrocoupling does not occur *via* stibinidene (*i.e.* RSb) elimination, which would only produce cyclic oligomers of the type [MesSb]_*n*_. Thus, we envisage deprotonation of MesSbH_2_ by Cp′_3_Y, which acts as a pre-catalyst, leading to the putative stibinide complex [Cp′_2_YSb(H)Mes], *i.e.* the monomeric unit of the trimer **1-Y**. Addition of a second equivalent of MesSbH_2_ can lead to the formation of a cyclic, four-membered transition state in which the distibine Sb_2_H_2_Mes_2_ forms, thus generating a hydride-ligated intermediate [Cp′_2_YH]. A second four-membered transition state can then be proposed from which [Cp′_2_YSb(H)Mes] is re-formed along with elimination of dihydrogen. The proposed σ-bond metathesis transition states in Scheme 2 are consistent with those thought to occur in several different types of dehydrocoupling reactions catalysed by main group and transition metal complexes.^42^ To account for the formation of Sb_4_Mes_4_ from Sb_2_H_2_Mes_2_, we propose a mechanism in which the distibine is deprotonated by [Cp′_2_YH] to give an intermediate distibinide complex [Cp′_2_Y{RSb–Sb(H)R}], which subsequently undergoes a β-hydride elimination to regenerate the yttrium hydride and form the distibene MesSb=SbMes. Since heavy p-block analogues of alkenes tend to cyclo-oligomerize owing to the weak nature of the multiple bonds,^43^ the formation of Sb_4_Mes_4_ can be accounted for by dimerization of the distibene.

Dehydrocoupling catalysis has emerged as one of the most important methods for the synthesis of homo- or hetero-nuclear bonds between p-block elements.^44^ Considerable attention has focused on the synthesis of inorganic polymers, especially poly(ammonia-borane) and poly(amine-boranes), owing to their proposed applications as hydrogen storage and delivery materials.^45^ Notably, only one example of metal-catalysed stibine dehydrocoupling has previously been reported, which employed the group 4 metallocenes [(Cp*)(Cp)M(H)Cl] (M = Zr, Hf) as catalysts at 5 mol% loading for the formation of Sb_4_Mes_4_ from MesSbH_2_.^46^ This reaction is thought to proceed *via* a mechanism that involves α-elimination of highly reactive stibinidene (SbR) fragments, which subsequently cyclo-oligomerize to Sb_*n*_R_*n*_. Many catalysts based on main group metals and transition metals are well established for the dehydrocoupling of a range of element–element bonds,^42,44^ however surprisingly few examples employ rare earth elements. A recent study has shown that divalent rare earth alkyl complexes are effective catalysts for the cross-dehydrocoupling of silanes and amines to give silazanes.^47^

The cross-dehydrocoupling of **1-Dy** with mesitylstibine to give **2-Dy** is the first example of such reactivity being used to synthesize an SMM. Our observations therefore represent a new catalytic transformation in rare-earth chemistry and a new synthetic strategy in molecular magnetism. The observation of SMM behaviour for **1-Dy**, **2-Dy** and their magnetically dilute analogues in light of the role of **1-Y** and **2-Y** in stibine dehydrocoupling is also significant. Although the paramagnetism of the dysprosium systems precludes detailed study by NMR spectroscopy, crystalline Sb_2_H_2_Mes_2_, Sb_4_Mes_4_ can be isolated from the dehydrocoupling of MesSbH_2_ catalysed by 10 mol% Cp′_3_Dy. In light of the similar chemistry of Y^3+^ and Dy^3+^, **1-Dy** and **2-Dy** should therefore also be intermediates in the catalytic stibine dehydrocoupling. Thus, the dysprosium–antimony compounds display two functions that can be accessed by varying the temperature, since cooling **1-Dy** and **2-Dy** below 40 K leads to SMM behaviour, and heating them in solution above 313 K results in catalytic stibine dehydrocoupling.

## Conclusions

In summary we have synthesized the first antimony-ligated SMMs. The anisotropy barriers of **1-Dy**, **2-Dy** in zero applied field, and of their diluted analogues, are *U*_eff_ = 345 cm^–1^ and 270 cm^–1^, respectively, placing them amongst the highest yet reported. The conversion of **1-Dy** into **2-Dy** *via* cross dehydrocoupling with mesitylstibine represents a novel synthetic strategy in molecular magnetism. Indeed, our initial aim of targeting SMMs with lanthanide–metalloid bonds has resulted in the identification new catalytic reactivity for the rare earth elements. Given the broad scope of dehydrocoupling chemistry, the synthetic strategy has considerable potential to be extended to incorporate many new and unconventional chemical environments into molecular magnets. The next challenge is to extend the reactivity to synthesize SMMs that can be regarded as molecular alloys, *i.e.* systems in which the magnetic centres are bonded to the heaviest stable metallic elements. Based on the periodic trend in the anisotropy barrier unearthed during this study, *i.e.* that *U*_eff_ tends to increase with increasing radius of the pnictogen, substantial increases in *U*_eff_ can be expected for SMMs ligated by the 6p elements thallium, lead and bismuth, provided the chemical environments can be stabilized. On-going work in our laboratory will pursue these targets.

## References

[cit1] Fataftah M. S., Zadrozny J. M., Coste S. C., Graham M. J., Rogers D. M., Freedman D. E. (2016). J. Am. Chem. Soc..

[cit2] Zheng Y.-Z., Zhou G.-J., Zheng Z., Winpenny R. E. P. (2014). Chem. Soc. Rev..

[cit3] Brooker S. (2015). Chem. Soc. Rev..

[cit4] Bousseksou A., Molnár G., Salmon L., Nicolazzi W. (2011). Chem. Soc. Rev..

[cit5] Blackburn O. A., Kenwright A. M., Jupp A. R., Goicoechea J. M., Beer P. D., Faulkner S. (2016). Chem.–Eur. J..

[cit6] Liu W.-M., Keizers P. H. J., Hass M. A. S., Blok A., Timmer M., Sarris A. J. C., Overhand M., Ubbink M. (2012). J. Am. Chem. Soc..

[cit7] Shen C., New E. J. (2013). Curr. Opin. Chem. Biol..

[cit8] Woodruff D. N., Winpenny R. E. P., Layfield R. A. (2013). Chem. Rev..

[cit9] Papatriantafyllopoulou C., Moushi E. E., Christou G., Tasiopoulos A. J. (2016). Chem. Soc. Rev..

[cit10] Gatteschi D., Sessoli R. and Villain J., Molecular Nanomagnets, Oxford University Press, Oxford, 2006.

[cit11] Frost J. M., Harriman K. L. M., Murugesu M. (2016). Chem. Sci..

[cit12] Zadrozny J. M., Xiao D. J., Atanasov M., Long G. J., Grandjean F., Neese F., Long J. R. (2013). Nat. Chem..

[cit13] Layfield R. A. (2014). Organometallics.

[cit14] Ishikawa N., Sugita M., Ishikawa T., Koshihara S., Kaizu Y. (2003). J. Am. Chem. Soc..

[cit15] AlDamen M. A., Clemente-Juan J. M., Coronado E., Martí-Gastaldo C., Gaita-Ariño A. (2008). J. Am. Chem. Soc..

[cit16] Zhang P., Zhang L., Wang C., Xue S. F., Lin S. Y., Tang J. (2014). J. Am. Chem. Soc..

[cit17] Rinehart J. D., Fang M., Evans W. J., Long J. R. (2011). J. Am. Chem. Soc..

[cit18] Layfield R. A., McDouall J. J. W., Sulway S. A., Collison D., Tuna F., Winpenny R. E. P. (2010). Chem.–Eur. J..

[cit19] Zhang P., Zhang L., Tang J. (2015). Dalton Trans..

[cit20] Chen Y.-C., Liu J.-L., Ungur L., Liu J., Li Q.-W., Wang L.-F., Ni Z.-P., Chibotaru L. F., Chen X.-M., Tong M.-L. (2016). J. Am. Chem. Soc..

[cit21] Ding Y.-S., Chilton N. F., Winpenny R. E. P., Zheng Y.-Z. (2016). Angew. Chem., Int. Ed..

[cit22] Gupta S. K., Rajeshkumar T., Rajaraman G., Murugavel R. (2016). Chem. Sci..

[cit23] Cervetti C., Rettori A., Pini M. G., Cornia A., Repollé A., Luis F., Dressel M., Rauschenbach S., Kern K., Burghard M., Bogani L. (2016). Nat. Mater..

[cit24] Thiele S., Balestro F., Ballou R., Klyatskaya S., Ruben M., Wernsdorfer W. (2014). Science.

[cit25] Ateş M., Breunig H. J., Güleç S., Offermann W., Häberle K., Dräger M. (1989). Chem. Ber..

[cit26] Balázs G., Breunig H. J., Lork E., Offerman W. (2001). Organometallics.

[cit27] Evans W. J., Gonzales S. L., Ziller J. W. (1992). J. Chem. Soc., Chem. Commun..

[cit28] Breunig H. J., Lork E., Moldovan O., Rat C. I., Rosenthal U., Silvestru C. (2009). Dalton Trans..

[cit29] Iwahara N., Chibotaru L. F. (2015). Phys. Rev. B: Condens. Matter Mater. Phys..

[cit30] Lines M. E. (1971). J. Chem. Phys..

[cit31] Chilton N. F., Anderson R. P., Turner L. D., Soncini A., Murray K. S. (2013). J. Comput. Chem..

[cit32] Pugh T., Chilton N. F., Layfield R. A. (2016). Angew. Chem., Int. Ed..

[cit33] Pointillart F., Bernot K., Gohlen S., Le Guennic B., Guizouarn T., Ouahab L., Cador O. (2015). Angew. Chem., Int. Ed..

[cit34] Aquilante F. (2016). J. Comput. Chem..

[cit35] Blagg R. J., Ungur L., Tuna F., Speak J., Comar P., Collison D., Wernsdorfer W., McInnes E. J. L., Chibotaru L. F., Winpenny R. E. P. (2013). Nat. Chem..

[cit36] Wu J., Jung J., Zhang P., Zhang H., Tang J., Le Guennic B. (2016). Chem. Sci..

[cit37] Oyarzabal I., Ruiz J., Ruiz E., Aravena D., Seco J. M., Colacio E. (2015). Chem. Commun..

[cit38] Costes J. P., Duhayon C., Titos-Padilla S., Colacio E., Oyarzabal I., Gupta T., Rajaraman G. (2016). Inorg. Chem..

[cit39] Gagliardi L., Lindh R., Karlström G. (2004). J. Chem. Phys..

[cit40] Pugh T., Tuna F., Ungur L., Collison D., McInnes E. J. L., Chibotaru L. F., Layfield R. A. (2015). Nat. Commun..

[cit41] Pugh T., Vieru V., Chibotaru L. F., Layfield R. A. (2016). Chem. Sci..

[cit42] Leitao E. M., Jurca T., Manners I. (2013). Nat. Chem..

[cit43] Power P. P. (1998). J. Chem. Soc., Dalton Trans..

[cit44] Waterman R. (2013). Chem. Soc. Rev..

[cit45] Bhunya S., Malakar T., Ganguly G., Paul A. (2016). ACS Catal..

[cit46] Waterman R., Tilley T. D. (2006). Angew. Chem., Int. Ed..

[cit47] Pindwal A., Ellern A., Sadow A. D. (2016). Organometallics.

