# Supplementary material for: Antimony-ligated dysprosium single-molecule magnets as catalysts for stibine dehydrocoupling
Source: Chem Sci. 2016 Nov 21;8(3):2073–80. doi: 10.1039/c6sc04465d (PMC5399632; doi:10.1039/c6sc04465d)
Supplement: Supplementary file 1 [file SC-008-C6SC04465D-s001.pdf]

Electronic Supplementary Material (ESI) for Chemical Science.
This journal is © The Royal Society of Chemistry 2016

Antimony-Ligated Single-Molecule Magnets as Catalysts for Stibine Dehydrocoupling

Thomas Pugh, Nicholas F. Chilton and Richard A. Layfield

School of Chemistry, The University of Manchester, Oxford Road, Manchester, M13 9PL, U.K.

Contents

1. Synthesis and crystallographic characterization 1-4
2. NMR and IR spectroscopy 6-6
3. Magnetic property measurements 7-10
4. Theoretical characterization 11-13
5. Catalytic stibine dehydrocoupling 14-16
6. References 17

1. Synthesis and crystallographic characterization

All manipulations were performed under an atmosphere of dry, oxygen-free argon, using either standard Schlenk techniques or an argon-filled glove box. Toluene and THF, and their deuterated analogues for NMR spectroscopy, were dried by refluxing over potassium and collected by distillation. All solvents were stored over activated 4 Å molecular sieves or a potassium mirror, and freeze-thaw degassed prior to use. Anhydrous rare earth chlorides (99.99% purity), *n*-butyllithium (1.6 M in hexanes), antimony(III) chloride, lithium aluminium hydride, mesitylmagnesium bromide (1.0 M in THF) and di-methylcyclopentadiene (95%) were purchased from Sigma-Aldrich. Mesitylstibine[1] and rare earth *tris*-(methyl)cyclopentadienide complexes were synthesised according to literature procedures.[1,2] Elemental analyses were carried out at London Metropolitan University, U.K. Infrared spectra were recorded in an inert atmosphere using a Bruker Alpha FT-IR spectrometer. X-ray diffraction data were collected on an Oxford Instruments XCalibur2 diffractometer or an Agilent SuperNova, using MoKα radiation. NMR spectra were acquired on a Varian VMS 500 MHz spectrometer.

[(η^5-Cp′$_2$Dy){μ-Sb(H)Mes}]$_3$·toluene (1-Dy·toluene). A solution of *n*-butyllithium in hexanes (1.6 M, 0.63 ml, 1.0 mmol) was added to a solution of Cp′$_3$Dy (0.400 g, 1.0 mmol) in toluene (6 ml) at –78°C, and the mixture was stirred for 0.5 hours. The pale suspension was warmed to –50°C and a solution of MesSbH$_2$ (0.242 g, 1.0 mmol) in toluene (4 ml) was added dropwise over 0.5 hours. The pale orange mixture was quickly warmed to room temperature, filtered immediately and concentrated *in vacuo* until copious precipitate formed. The precipitate was re-dissolved with gentle heating and the solution was stored at 4°C overnight, which resulted in the formation of **1-Dy**·toluene as pale orange crystals (0.293 g, 50% isolated yield). Elemental analysis, found/% (calculated/%) for **1-Dy**·toluene: C 47.14 (47.23), H 4.85 (4.87).

[(η^5-Cp′$_2$Dy)$_3${μ-(SbMes)$_3$Sb}]·toluene (2-Dy·toluene). A solution of *n*-butyllithium in hexanes (1.6 M, 0.39 ml, 0.62 mmol) was added to a solution of Cp′$_3$Dy (0.248 g, 0.6 mmol) in toluene (6 ml) at –78°C, and the mixture was stirred for 0.5 hours. At –78°C, a toluene solution (4 ml) of MesSbH$_2$ (0.200 g, 0.83 mmol) was added dropwise over 0.5 hours to the resulting pale-yellow suspension, and the reaction mixture was slowly warmed to room temperature (about 21°C) over a period of 12 h. The dark red reaction mixture was filtered and concentrated *in vacuo* until copious precipitate formed. The precipitate was re-dissolved with gentle heating and the solution was stored at 4°C overnight, which resulted in the formation of **2-Dy**·toluene as dark red crystals (0.226 g, 57% isolated yield). Elemental analysis, found/% (calculated/%) for **2-Dy**·toluene: 44.18 (44.27), H 4.38 (4.41).

[(η^5-Cp'_2Y){*μ*-Sb(H)Mes}]$_3$·toluene (1-Y·toluene). The synthesis was performed using the method described above for **1-Dy**·toluene, using Cp'_3Y (0.326 g, 1.0 mmol), *n*-butyllithium in hexanes (0.63 ml, 1.0 mmol) and $MesSbH_2$ (0.242 g, 1.0 mmol). **1-Y**·toluene was isolated as pale orange crystals (0.268 g, 52 %). Elemental analysis, found/% (calculated/%) for **1-Y**: 53.87 (53.91), H 5.54 (5.56). 1H NMR (toluene-D8, 298.15 K, *δ*/ppm): 6.95-6.03 (mesityl and Cp′ CH resonances); 2.70-1.44 (mesityl and Cp′ CH3 resonances). The multiple different resonances for the similar environments stem from the chemical equivalence but magnetic inequivalence, which is due to restricted rotation of the various substituents.

[(η^5-Cp'_2Y)$_3${*μ*-$(SbMes)_3Sb$}]·toluene (2-Y·toluene**).** The synthesis was performed using the method described above for **2-Dy**·toluene, using Cp'_3Y (0.202 g, 0.6 mmol), *n*-butyllithium in hexanes (0.39 ml, 0.6 mmol) and SbH_2Mes (0.200 g, 0.8 mmol). **2-Y**·toluene was isolated as dark red crystals (0.172 g, 50%). Elemental analysis, found/% (calculated/%) for **2-Y**·toluene: 50.16 (50.10), H 4.91 (4.99). ^{1}H NMR (toluene-D_8, 298.15 K, *δ*/ppm): 6.82 (s, 3H, *meta*-C$\underline{H}$), 6.76 (s, 3H, *meta*-C$\underline{H}$), 6.22 (t, 6H, Cp-$\underline{H}$), 6.41 (t, 6H, Cp-$\underline{H}$), 5.81 (t, 6H, Cp-$\underline{H}$), 5.71 (t, 6H, Cp-$\underline{H}$), 3.12 (s, 9H, *ortho*-C$\underline{H}_3$), 2.41 (s, 9H, *ortho*-C$\underline{H}_3$), 2.30 (s, 9H, Cp-C$\underline{H}_3$), 2.13 (s, 9H, para-C$\underline{H}_3$), 1.62 (s, 9H, Cp-C$\underline{H}_3$). ^{13}C NMR (toluene-d_8, 298.15 K, δ/ppm): 145.50 (*ortho*-$\underline{C}$-CH_3), 140.32 (*ortho*-$\underline{C}$-CH_3), 135.91 (*para*-$\underline{C}$-CH_3), 129.43 (*meta*-$\underline{C}$-H), 128.37 (*meta*-$\underline{C}$-H), 123.29 (Cp-$\underline{C}$-CH_3), 122.68 (Cp-$\underline{C}$-CH_3), 112.69 (Cp-$\underline{C}$H), 111.53 (Cp-$\underline{C}$H), 111.39 (Cp-$\underline{C}$H), 109.51 (Cp-$\underline{C}$H).

Synthesis of 2-Y from 1-Y. $MesSbH_2$ (2.4 mg, 0.01 mmol) and C_6D_6 (0.6 ml) were added to a J. Youngs NMR tube containing **1-Y (**15.6 mg, 0.01 mmol). After 10 days at room temperature, the ^{1}H spectrum of the reaction mixture showed that all resonances attributed to **1-Y** had been replaced by those of **2-Y**, and H_2 formation was observed (Figure S5).

Synthesis of 2-Dy from 1-Dy. $MesSbH_2$ (4.1 mg, 0.017 mmol) and toluene (0.5 ml) were added to a vial containing **1-Dy (**30.0 mg, 0.017 mmol). After 10 days at room temperature, hexane was added to the darkened reaction mixture and an orange precipitate was collected by filtration (14.4 mg, 45%). The IR spectrum of the material was identical to that of **2-Dy** as synthesized according to the procedure on p1.

Synthesis of Dy@1-Y·toluene. The synthesis was performed using the method described above for **1-Dy**·toluene and **1-Y**·toluene using Cp'_3Y (0.257 g, 0.79 mmol), Cp'_3Dy (0.017 g, 0.04 mmol) *n*-butyllithium in hexanes (1.6 M, 0.52 ml, 0.83 mmol) and $MesSbH_2$ (0.200, 0.83 mmol). **Dy@1-Y**·toluene was isolated as pale orange crystals (0.212 g, 52 % isolated yield based on antimony).

Synthesis of Dy@2-Y·toluene. The synthesis was performed using the method described above for **2-Dy**·toluene and **2-Y**·toluene using Cp'_3Y (0.310 g, 0.95 mmol), Cp'_3Dy (0.020 g, 0.05 mmol) butyllithium in hexanes (0.39 ml, 0.62 mmol) and SbH_2Mes (0.32 g, 1.33 mmol). **Dy@2-Y**·toluene was isolated as dark red crystals (0.257 g, 46 % isolated yield based on antimony).

Characterization of doped materials

The two doped materials were characterized by single crystal X-ray diffraction. Full collections of several crystals of **Dy@1-Y**·toluene and **Dy@2-Y**·toluene were performed on an Oxford Xcaliber-2 diffractometer using Mo-Kα radiation at 100 K. Accurate dysprosium:yttrium ratios were measured by inductively coupled plasma atomic emission (ICP) spectroscopy using a Thermo iCap 6300 ICP-OES instrument, which resulted in dysprosium contents of 5.0±0.5% for both doped materials.

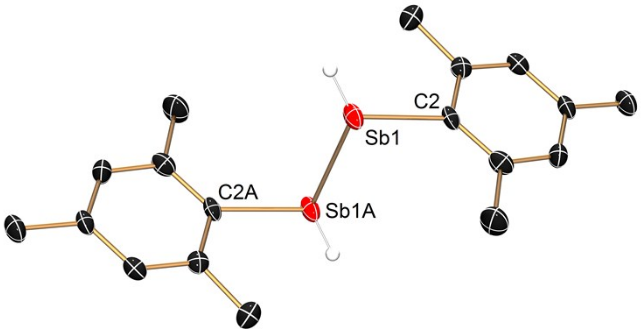

Figure S1. Thermal ellipsoid (50% probability) representation of the molecular structure of $Sb_2H_2Mes_2$. Hydrogen atoms, except those bonded to Sb, are omitted for clarity. Selected bond lengths (Å) and angles (°): Sb(1)–Sb(1A) 2.859(4), Sb(1)–C(2) 2.183(3), C(2)-Sb(1)-Sb(1A) 91.6(1), C(2)-Sb(1)-Sb(1A)-C(2A) 180.0(1).

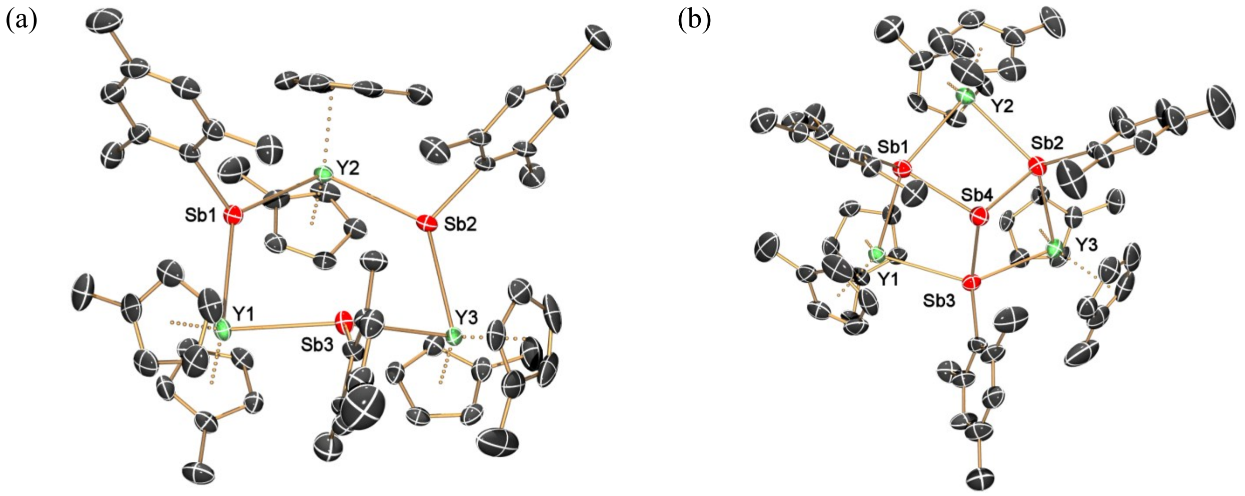

Figure S2. Thermal ellipsoid (50% probability) representation of the molecular structure of (a) **1-Y**. (b) Molecular structure of **2-Y**. Hydrogen atoms are omitted for clarity.

Table S1. Crystal data and structure refinement details.

	**1-Dy·toluene**	**2-Dy·toluene**	**1-Y·toluene**	**2-Y·toluene**	**$Sb_2H_2Mes_2$**
empirical formula	$C_{70}H_{83}Sb_3Dy_3$	$C_{70}H_{83}Sb_4Dy_3$	$C_{70}H_{83}Sb_3Y_3$	$C_{70}H_{83}Sb_4Y_3$	$C_{18}H_{24}Sb_2$
formula weight	1777.11	1898.86	1556.34	1678.09	483.87
temperature / K	150.01(10)	150.02(10)	150.02(11)	150.02(10)	150.01(13)
crystal system	monoclinic	monoclinic	monoclinic	monoclinic	triclinic
space group	*Cc*	Cc	Cc	Cc	P-1
a / Å	15.1130(2)	14.8968(3)	15.1052(2)	14.8643(4)	5.1740(2)
b / Å	18.7437(2)	19.3308(4)	18.7715(2)	19.3840(3)	7.9835(4)
c / Å	24.0965(3)	24.2616(5)	24.1263(3)	24.2728(7)	11.2694(5)
α / °	90	90	90	90	88.921(4)
β / °	103.3390(10)	106.568(2)	103.4370(10)	107.004(3)	79.679(4)
γ / °	90	90	90	90	73.714(4)
volume / $Å^3$	6641.75(14)	6696.5(2)	6653.68(14)	6688.0(3)	439.35(4)
Z	4	4	4	4	1
ρ_{calc} / mg mm^{-3}	1.777	1.883	1.554	1.667	1.829
crystal size / mm^3	0.2 × 0.2 × 0.1	0.2 × 0.2 × 0.1	0.2 × 0.2 × 0.2	0.1 × 0.1 × 0.05	0.2 × 0.1 × 0.02
2θ range/°	6.788 to 50.692	6.738 to 52.744	6.78 to 52.744	6.738 to 50.700	7.354 to 52.738
reflections collected	74897	46304	135283	25440	5797
independent reflections	12154	13695	13580	11082	1798
R(int)	0.0502	0.0561	0.0410	0.0540	0.0309
data/restraints/parameters	12154/377/853	13695/455/751	13580/259/878	11082/394/751	1798/0/112
goodness-of-fit on F^2	1.027	1.016	1.031	1.009	1.049
final R indexes [I≥2σ(I)]	$R_1 = 0.0282$, $wR_2 = 0.0635$	$R_1 = 0.0349$ $wR_2 = 0.0581$	$R_1 = 0.0213$ $wR_2 = 0.0445$	$R_1 = 0.0459$ $wR_2 = 0.0773$	$R_1 = 0.0236$, $wR_2 = 0.0516$
final R indexes [all data]	$R_1 = 0.0303$ $wR_2 = 0.0648$	$R_1 = 0.0411$, $wR_2 = 0.0615$	$R_1 = 0.0241$ $wR_2 = 0.0455$	$R_1 = 0.0693$ $wR_2 = 0.0868$	$R_1 = 0.0308$, $wR_2 = 0.0548$
largest diff. peak, hole/e.A^{-3}	3.56, –1.11	0.86/-0.66	1.06/-0.22	0.73/-0.66	0.37/-0.35
CCDC ref. code	1484572	1484571	1484573	1484570	1485316

Table S2. Selected bond lengths (Å) and angles (°) for **1-Dy**, **2-Dy**, **1-Y** and **2-Y**.

	**1-Dy**	**2-Dy**	**1-Y**	**2-Y**
$M–Sb_{range}$	3.092(6)-3.212(3)	3.119(1)-3.138(1)	3.1307(6)-3.2009(6)	3.121(2)-3.142(2)
$M–Sb_{av}$	3.166(6)	3.130(1)	3.1744(6)	3.134(2)
$M–C_{range}$	2.59(1)-2.651(9)	2.58(1)-2.66(1)	2.604(5)-2.649(5)	2.58(2)-2.66(2)
$M–C_{av}$	2.62(3)	2.62(1)	2.621(5)	2.62(2)
$M–Cp_c$	2.340(5)	2.340(6)	2.335(2)	2.332(7)
Cp_c-M-Cp_c	129.30(17)-130.92(19)	129.9(2)-130.3(2)	129.38(7)-130.77(9)	129.6(2)-130.6(2)
M---M	5.7174(7)-5.8535(5)	5.7175(8)-5.8293(8)	5.7332(7)-5.8643(5)	5.7296(19)-5.8393(18)
Sb-M-Sb	85.9(1)-103.85(7)	85.32(3)-89.15(3)	86.99(2)-102.64(7)	84.40(4)-88.14(4)
M-Sb-M	128.26(3)-136.73(14)	132.03(3)-136.96(3)	128.59(2)-137.12(5)	132.24(4)-137.04(5)
Sb–Sb	-	2.8583(11)-2.8687(11)	-	2.8514(14)-2.8622(13)

Figure S3. ^1H NMR spectrum of **1-Y** in toluene-D_8 at 298 K. The C_1 symmetry of **1-Y** in the solid state is reflected in the ^1H NMR spectrum, which shows multiple resonances for the inequivalent CH and CH_3 groups.

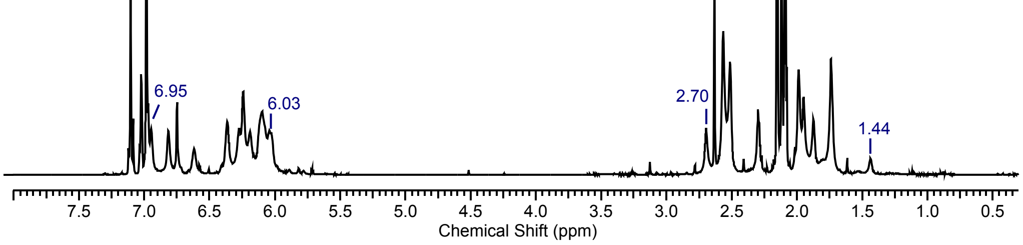

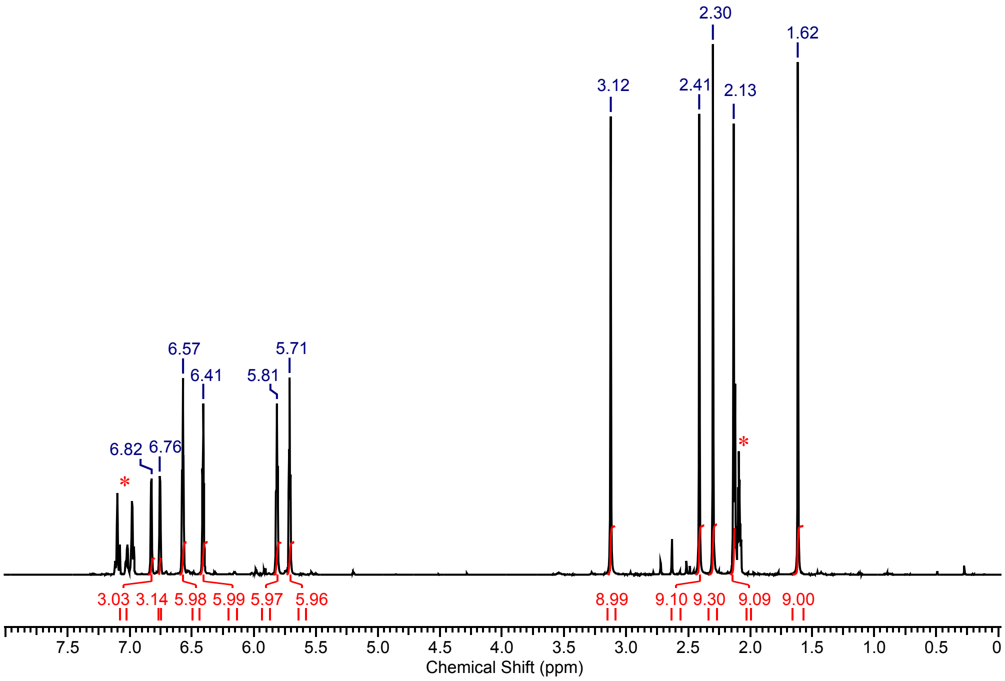

Figure S4. ^{1}H NMR spectrum of **2-Y** in toluene-D_8 at 298 K. The C_1 symmetry of **2-Y** in the solid state is reflected in the ^{1}H NMR spectrum, which shows multiple resonances for the inequivalent CH and CH_3 groups.

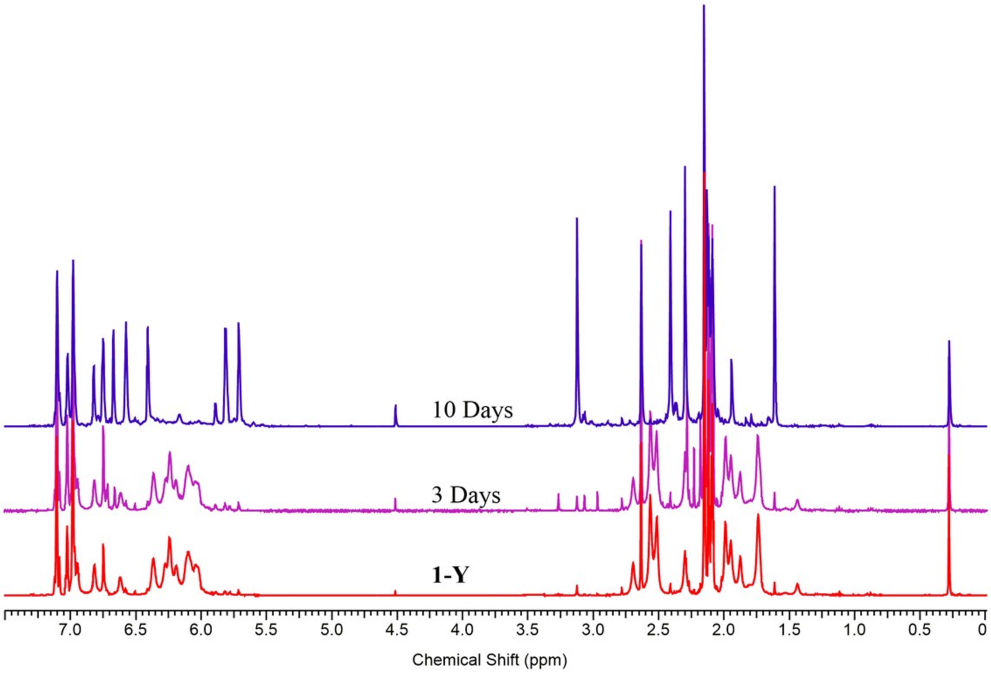

Figure S5. ^{1}H NMR spectrum of the reaction of **1-Y** with $MesSbH_2$ in to give **2-Y** (21°C, toluene-D_8).

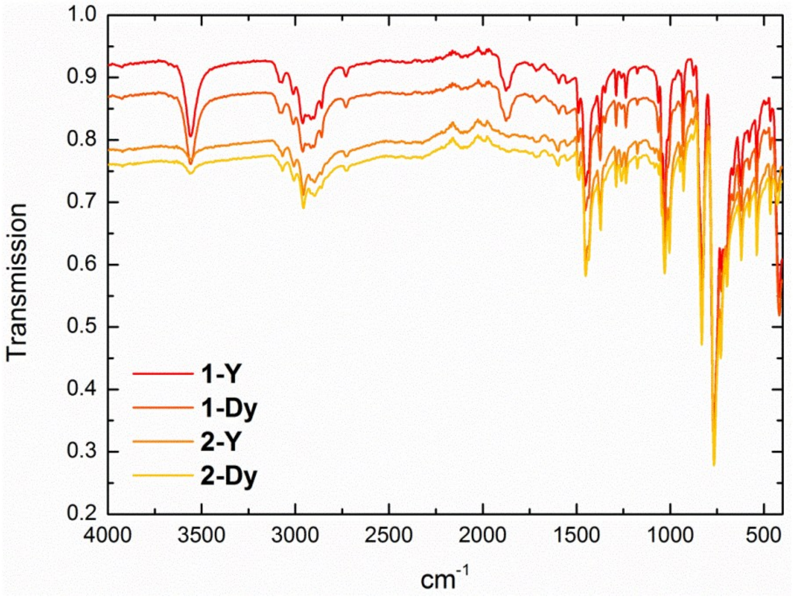

Figure S6. Infrared spectra of **1-Y**·toluene ($\tilde{\nu}_{\text{Sb–H}}$ = 1861, 1875 cm^{-1}), **1-Dy**·toluene ($\tilde{\nu}_{\text{Sb–H}}$ = 1860, 1873 cm^{-1}), **2-Y**·toluene and **2-Dy**·toluene.

Magnetic property measurements

The magnetic properties of polycrystalline samples of **1-Dy**·toluene, **2-Dy**·toluene, **Dy@1-Y**·toluene and **Dy@2-Dy**·toluene were measured using a Quantum Design MPMS-7 SQUID magnetometer at temperatures in the range 1.8-300 K. In a glove box, the polycrystalline samples were transferred to NMR tubes, restrained in eicosane and flame sealed under vacuum. Corrections for diamagnetism were made using Pascal's constants.

The magnetic susceptibility and magnetization of **1-Dy** and **2-Dy** were simulated by implementing the Lines model and the Hamiltonian shown below using the PHI software.

$$\hat{H} = \sum_{i=1}^{3} \sum_{k=2,4,6} \sum_{q=-k}^{k} B_{ki}^{q} \hat{O}_{ki}^{q} + \mu_B \sum_{i=1}^{3} g_J \vec{\hat{J}}_i \cdot \vec{B} - 2J_{iso}\left(\vec{\hat{S}}_1 \cdot \vec{\hat{S}}_2 + \vec{\hat{S}}_2 \cdot \vec{\hat{S}}_3 + \vec{\hat{S}}_3 \cdot \vec{\hat{S}}_1\right) \qquad \text{(S1)}$$

Here, the $\hat{O}_{ki}^{q}$ operator equivalents act on the $|J,m_J\rangle_i$ basis of the $^6H_{15/2}$ term of each Dy^{3+} ion where the B_{ki}^{q} crystal field terms are fixed from CASSCF calculations (see below) taking into account the relative orientations of the local reference frames of each Dy^{3+} ion. The only variable is the single isotropic Lines exchange constant J_{iso}, which acts on the true S = 5/2 spins of the Dy^{3+} ions via a Clebsch-Gordan decoupling. Modelling the interactions in this way, the best simulations are obtained for **1-Dy** and **2-Dy** using J_{iso} = –0.121 cm^{-1} and –0.150 cm^{-1}, respectively (Figures S7, S8).

(a) (b)

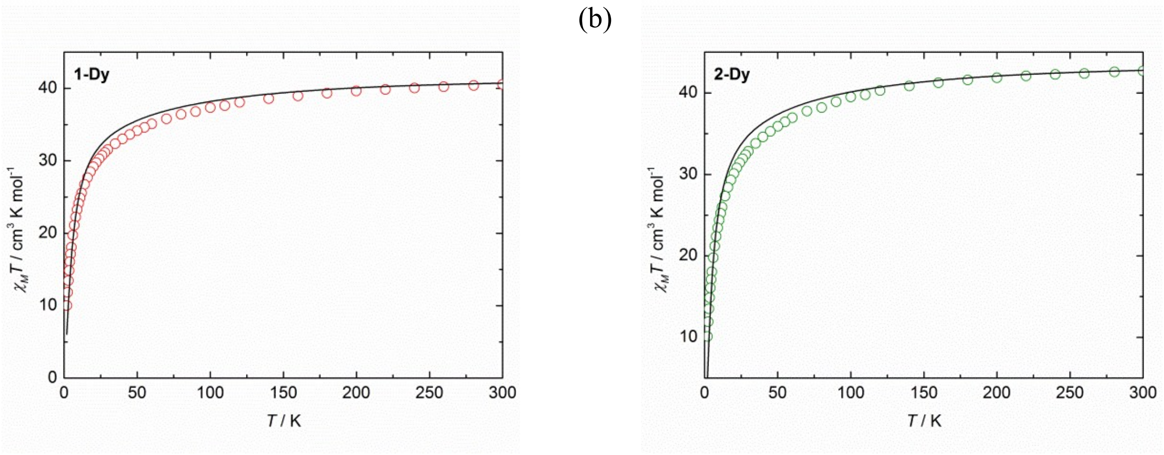

Figure S7. The product of the molar magnetic susceptibility with temperature ($\chi_M T$) against temperature for: (a) **1-Dy**·toluene; (b) **2-Dy**·toluene. Measurements were made using an applied field of 10 kOe. In both graphs, the experimental data (circles) are compared with the CASSCF results (solid lines), where the latter have been scaled by factors of 0.975 and 1.025 to match experiment.

(a) (b)

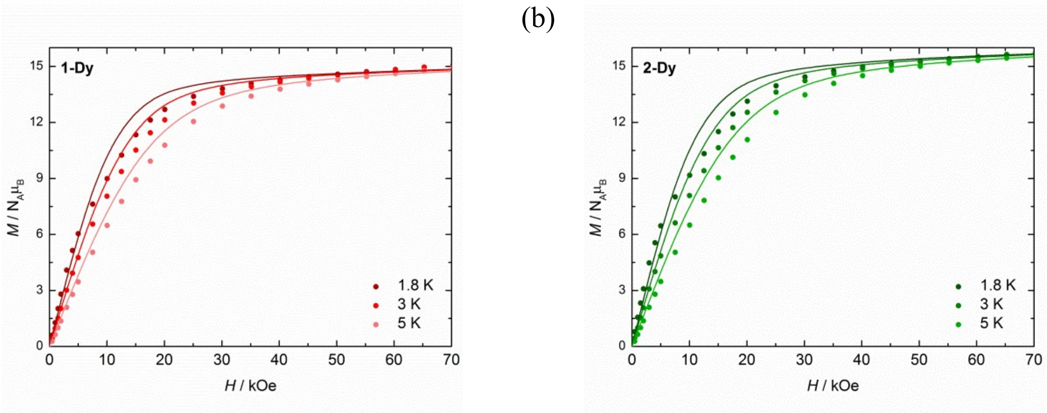

Figure S8. Field dependence of the magnetization for **1-Dy**·toluene (left) and **2-Dy**·toluene (right) at 1.8K, 3K and 5K for: (a) **1-Dy**·toluene; (b) **2-Dy**·toluene. In both graphs, the experimental data (circles) are compared with the CASSCF results (solid lines), where the latter have been scaled by factors of 0.975 and 1.025 to match experiment.

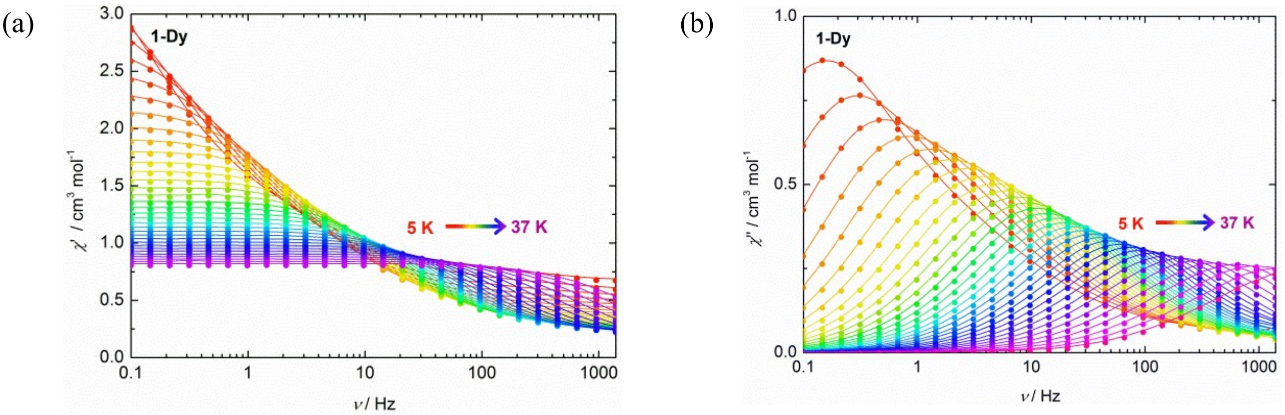

Figure S9. Frequency dependence of: (a) the in-phase (χ'), and; (b) the out-of-phase (χ'') magnetic susceptibility for **1-Dy**·toluene. An oscillating field of H_{ac} = 1.55 Oe was used with zero applied field.

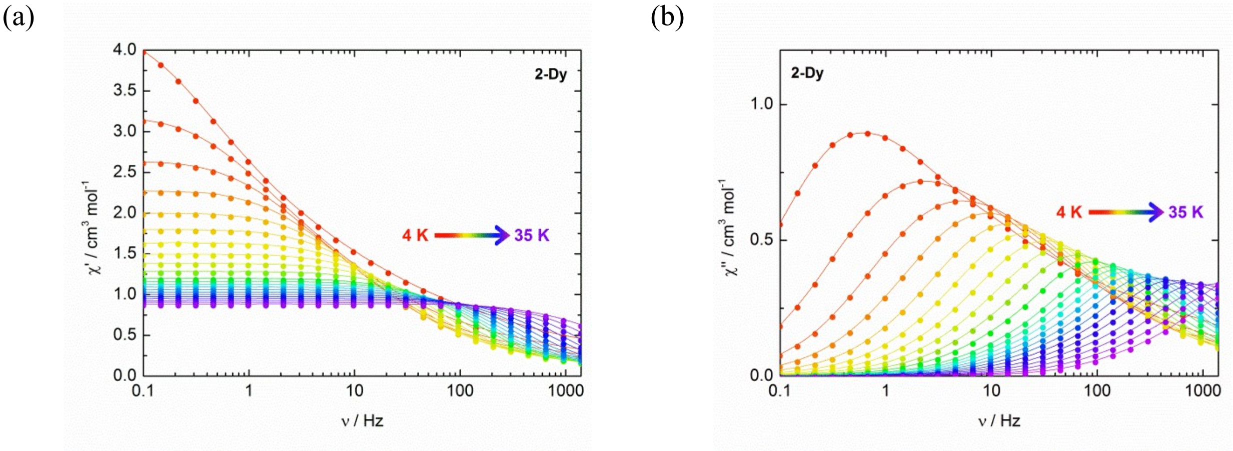

Figure S10. Frequency dependence of: (a) the in-phase (χ'), and; (b) the out-of-phase (χ'') magnetic susceptibility for **2-Dy**·toluene. An oscillating field of H_{ac} = 1.55 Oe was used with zero applied field.

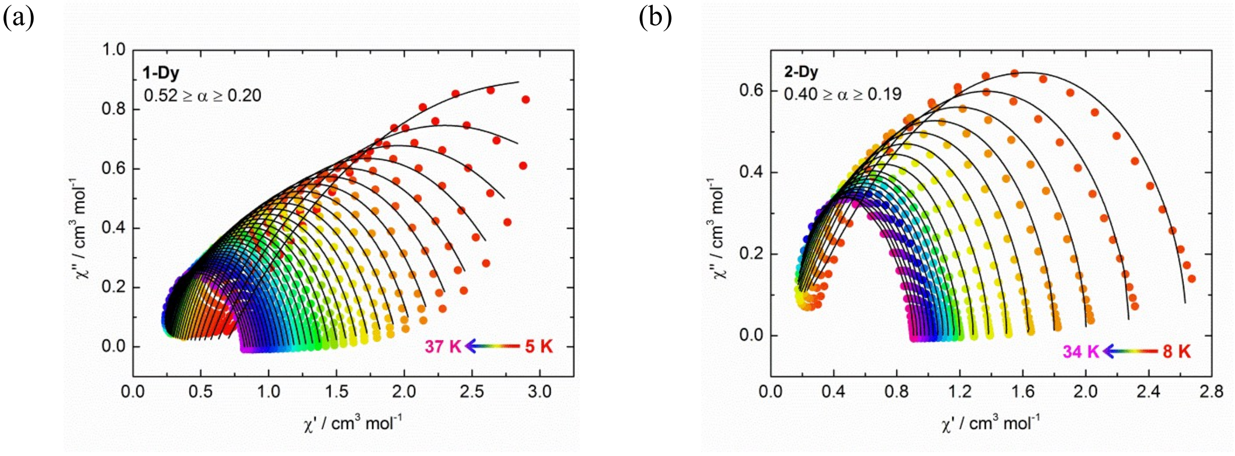

Figure S11. Argand (Cole-Cole) plots of χ'' *vs*. χ') for: (a) **1-Dy**·toluene, and; (b) **2-Dy**·toluene.

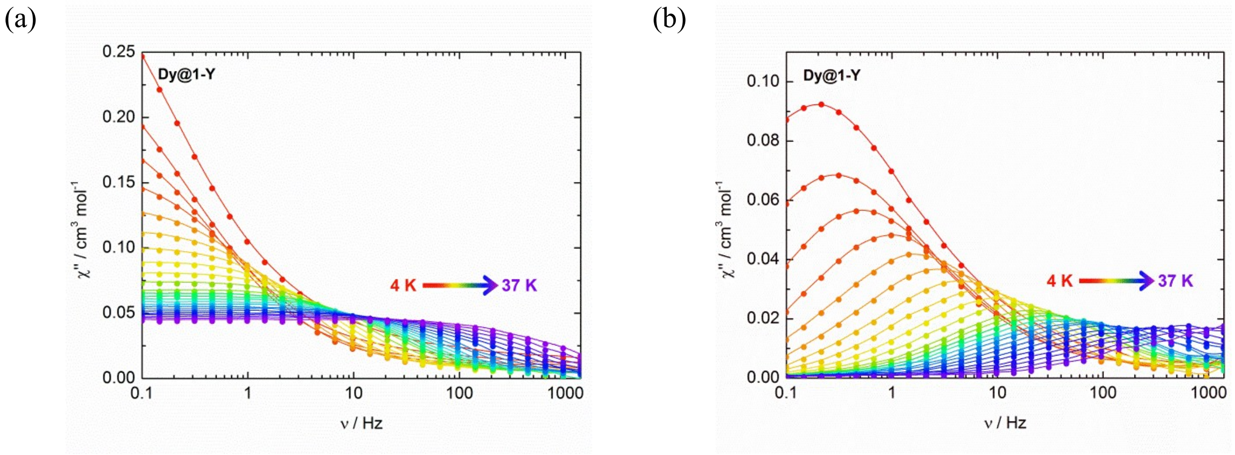

Figure S12. Frequency dependence of: (a) the in-phase (χ'), and; (b) the out-of-phase (χ'') magnetic susceptibility for **Dy@1-Y**·toluene. An oscillating field of H_{ac} = 1.55 Oe was used with zero applied field.

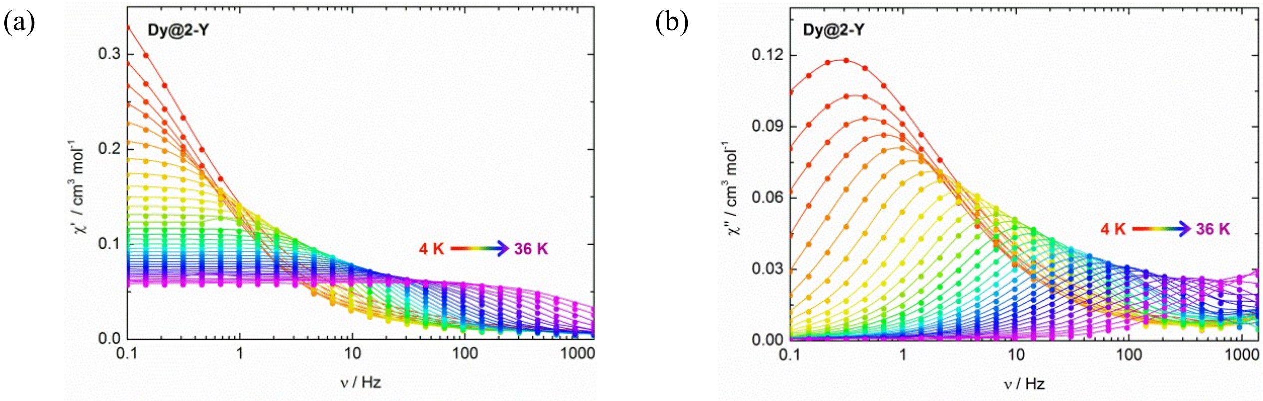

Figure S13. Frequency dependence of: (a) the in-phase (χ'), and; (b) the out-of-phase (χ'') magnetic susceptibility for **Dy@2-Y**·toluene. An oscillating field of H_{ac} = 1.55 Oe was used with zero applied field.

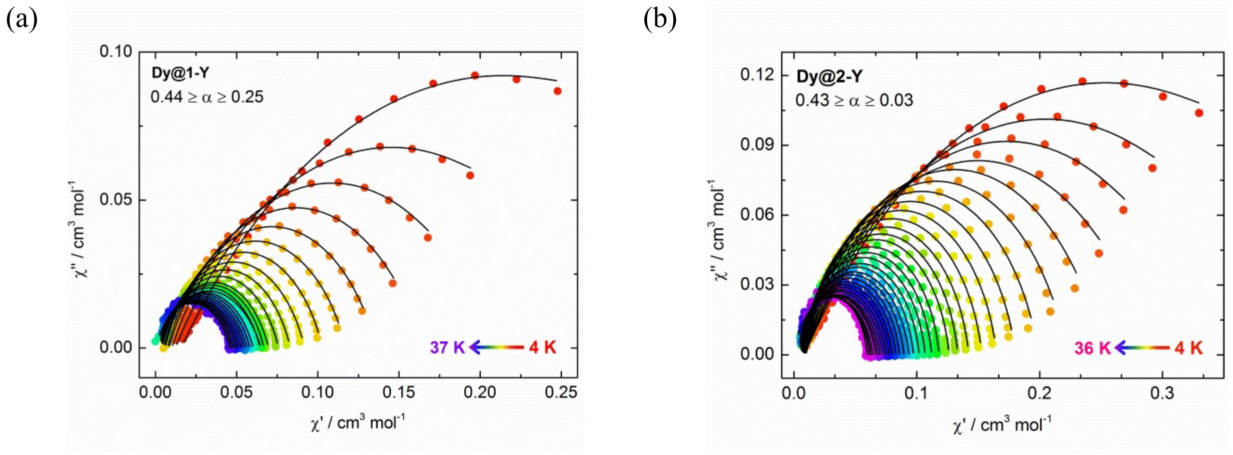

Figure S14. Argand (Cole-Cole) plots of χ'' *vs*. χ') for: (a) **Dy@1-Y**·toluene, and; (b) **Dy@2-Y**·toluene.

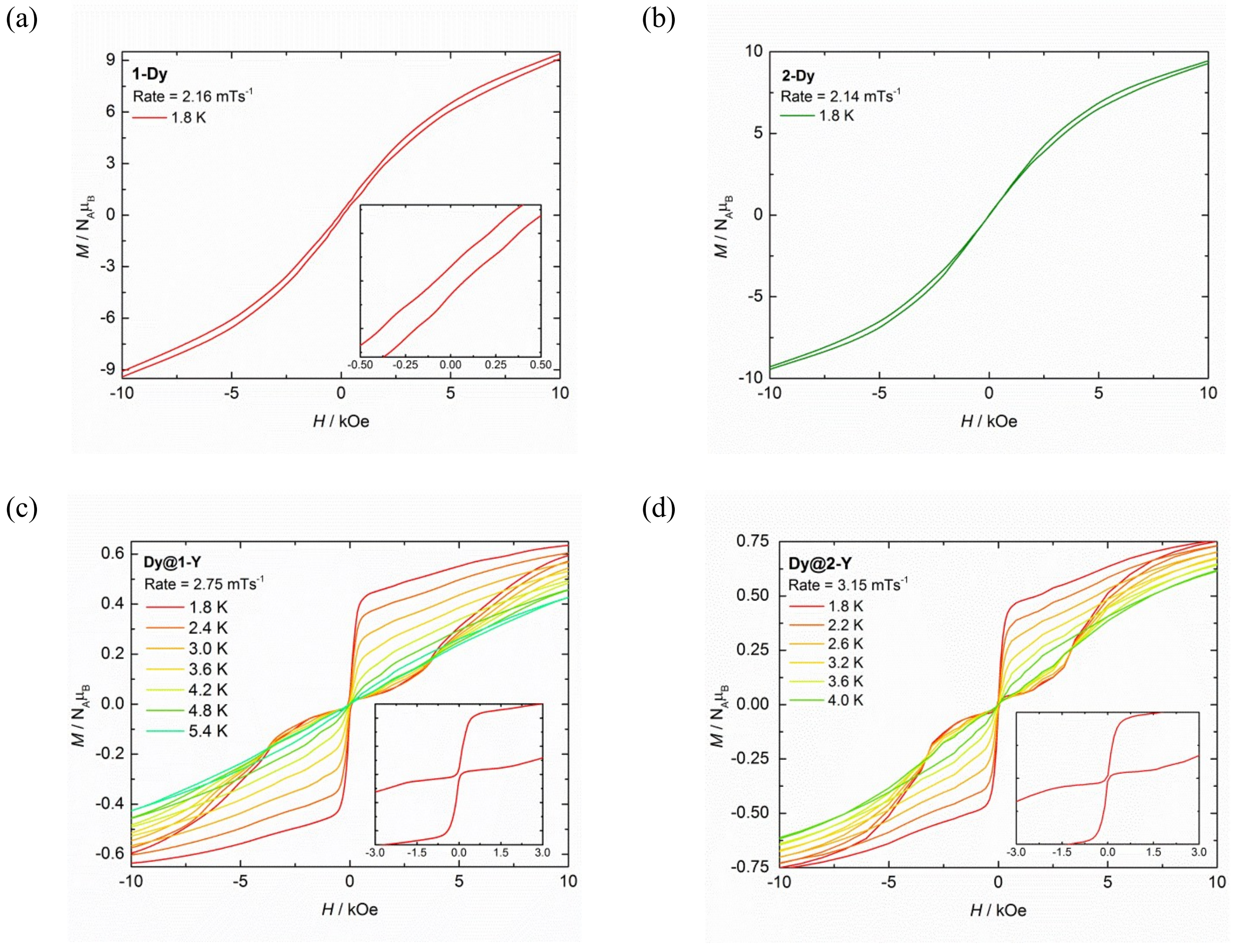

Figure S15. Magnetization (*M*) vs. field (*H*) hysteresis for (a) **1-Dy**·toluene; (b) **2-Dy**·toluene; (c) **Dy@1-Y**·toluene; (d) **Dy@2-Y**·toluene.

Theoretical characterization

CASSCF calculations were performed with MOLCAS 8.0[3] utilizing the solid-state geometry from X-ray crystallography. The H-atoms associated with the bridging antimony atoms could not be located experimentally for **1-Dy**, and therefore their positions were determined by DFT optimization with the remaining structure fixed. This optimization was performed with ORCA 3.0.2[4] using the BP86 functional,[5,6] the def2-TVP basis set for all atoms,[7,8] the dispersion corrections according to Grimme[9] and substitution of dysprosium atoms for yttrium to ensure SCF convergence.[10] The CASSCF calculations were performed for each Dy^{3+} centre independently, where the other Dy^{3+} centres were replaced with diamagnetic Lu^{3+}. The calculations employed the ANO-RCC basis sets using VTZP quality for the Dy^{3+} of interest, the antimony atoms, the coordinated Cp′ ligands, and MB quality for the other atoms. Cholesky decomposition of the two-electron integrals was performed using a threshold of 5×10^{-8}. The active space for the dysprosium calculations was the nine 4f electrons in the seven 4f orbitals, where 21, 224 and 490 roots of sextet, quartet and doublet multiplicity, respectively, were mixed by spin-orbit coupling in the state interaction step.

Table S3. Spin-orbit states for Dy1 in **1-Dy.**

Doublet	Energy / cm^{-1}	g_x	g_y	g_z	Angle / °
1	0	0.00	0.00	19.63	
2	170	0.00	0.00	17.11	2.5
3	324	0.01	0.02	14.74	4.5
4	413	0.45	0.48	11.68	3.5
5	486	2.34	3.45	7.68	5.4
6	504	0.02	1.11	18.27	89.8
7	534	3.31	7.73	10.54	89.0
8	638	0.01	0.10	19.16	89.7

Table S4. Spin-orbit states for Dy2 in **1-Dy.**

Doublet	Energy / cm^{-1}	g_x	g_y	g_z	Angle / °
1	0	0.00	0.00	19.54	
2	163	0.00	0.00	17.01	3.4
3	330	0.04	0.05	14.77	3.0
4	419	2.23	5.42	9.65	5.1
5	438	1.08	4.44	12.58	87.2
6	491	4.90	5.60	9.32	82.8
7	553	1.16	1.87	15.63	86.5
8	703	0.02	0.07	19.56	90.0

Table S5. Spin-orbit states for Dy3 in **1-Dy.**

Doublet	Energy / cm^{-1}	g_x	g_y	g_z	Angle / °
1	0	0.00	0.00	19.55	
2	168	0.00	0.00	16.98	2.0
3	334	0.07	0.07	14.71	2.9
4	418	2.58	4.71	12.47	62.1
5	437	2.09	5.12	8.22	58.4
6	493	4.99	5.98	9.33	80.7
7	557	0.91	1.50	15.83	87.7
8	721	0.02	0.06	19.61	89.8

Table S6. Spin-orbit states for Dy1 in **2-Dy.**

Doublet	Energy / cm^{-1}	g_x	g_y	g_z	Angle / °
1	0	0.00	0.00	19.66	
2	165	0.00	0.00	17.14	3.6
3	316	0.05	0.07	14.64	2.0
4	404	0.94	1.02	11.41	4.7
5	476	3.19	5.29	6.64	25.1
6	507	0.22	2.52	15.74	87.4
7	530	3.57	6.08	11.88	83.0
8	625	0.05	0.10	19.11	89.3

Table S7. Spin-orbit states for Dy2 in **2-Dy.**

Doublet	Energy / cm^{-1}	g_x	g_y	g_z	Angle / °
1	0	0.00	0.00	19.64	
2	167	0.00	0.00	17.09	2.8
3	333	0.02	0.02	14.74	1.1
4	431	0.30	0.44	11.71	3.4
5	486	0.74	0.94	19.20	87.7
6	501	4.08	5.62	7.62	16.3
7	548	2.30	4.00	13.79	87.4
8	678	0.01	0.11	19.48	89.5

Table S8. Spin-orbit states for Dy3 in **2-Dy.**

Doublet	Energy / cm^{-1}	g_x	g_y	g_z	Angle / °
1	0	0.00	0.00	19.67	
2	167	0.00	0.00	17.13	2.6
3	324	0.02	0.02	14.70	1.5
4	416	0.54	0.57	11.59	3.6
5	492	1.92	3.27	7.29	12.8
6	514	0.46	1.93	17.13	86.3
7	544	3.56	7.81	10.21	82.2
8	640	0.02	0.13	19.11	89.5

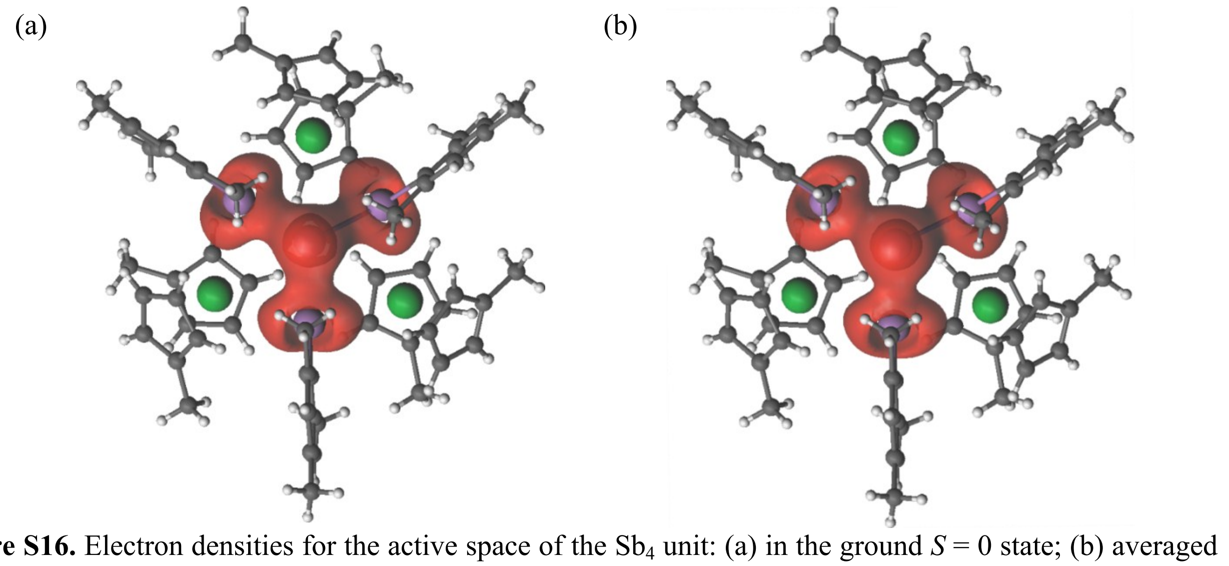

Figure S16. Electron densities for the active space of the Sb_4 unit: (a) in the ground $S = 0$ state; (b) averaged over the ten excited $S = 1$ states.

Table S9. Spin-orbit states of the Sb_4 unit.

State	Energy (cm^{-1})	State	Energy (cm^{-1})
1	0	21	37255.94
2	25962.35	22	37958.47
3	26024.42	23	38124.04
4	26032.12	24	40142.88
5	29413.15	25	40289.99
6	29601.61	26	40511.11
7	29694.07	27	40888.1
8	29839.08	28	41001.02
9	30089.54	29	41188.17
10	30227.85	30	41270.03
11	33124.43	31	41473.57
12	33128.6	32	41713.13
13	33180.78	33	41918.29
14	34956.11	34	42081.65
15	35242.48	35	43805.76
16	35263.46	36	44056.65
17	35404.29	37	44075.23
18	35566.32	38	44329.46
19	35625.38	39	44859.01
20	35833.19	40	44960.81

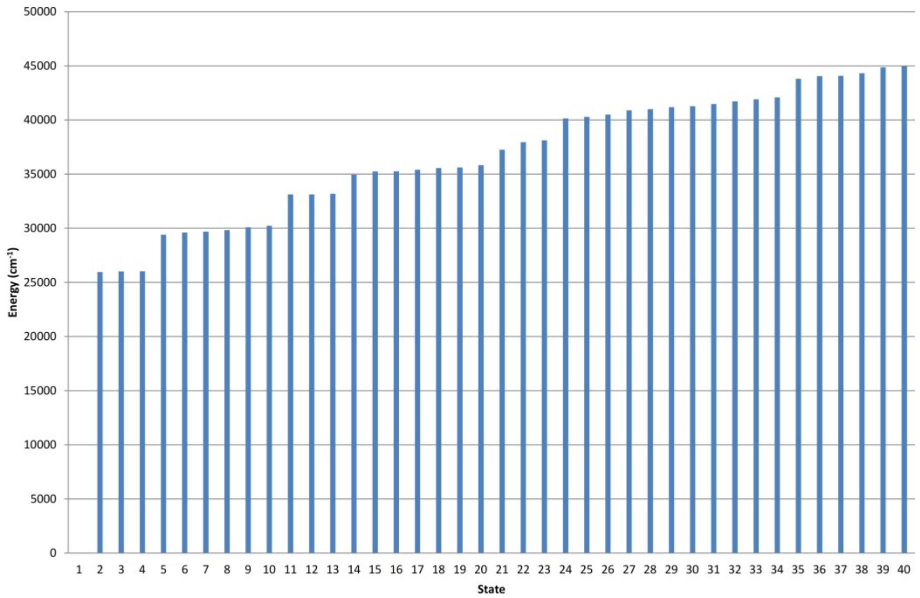

Figure S17. Spin-orbit state energies for the Sb_4 unit.

Catalytic stibine dehydrocoupling

For catalytic reactions, 0.1 mM solutions of $MesSbH_2$ and catalyst were prepared using a microbalance and a volumetric flask. Aliquots of both catalyst and substrate were taken and mixed in a J. Young NMR tube before rapidly being transferred to the NMR spectrometer. All spectra were acquired in toluene-D_8 solution.

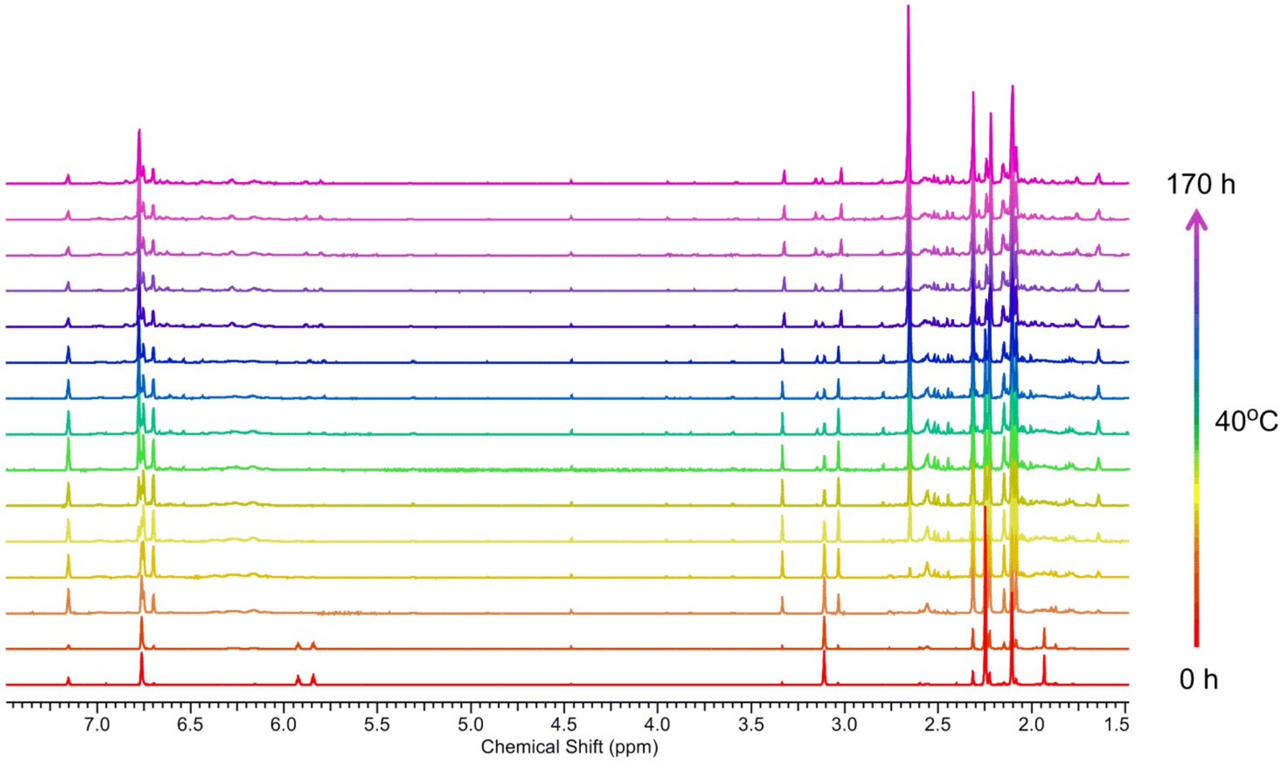

Figure S18. Variable-time ^{1}H NMR spectrum of the dehydrocoupling of $MesSbH_2$ catalysed by 10 mol% Cp'_3Y. Temperature = 40°C.

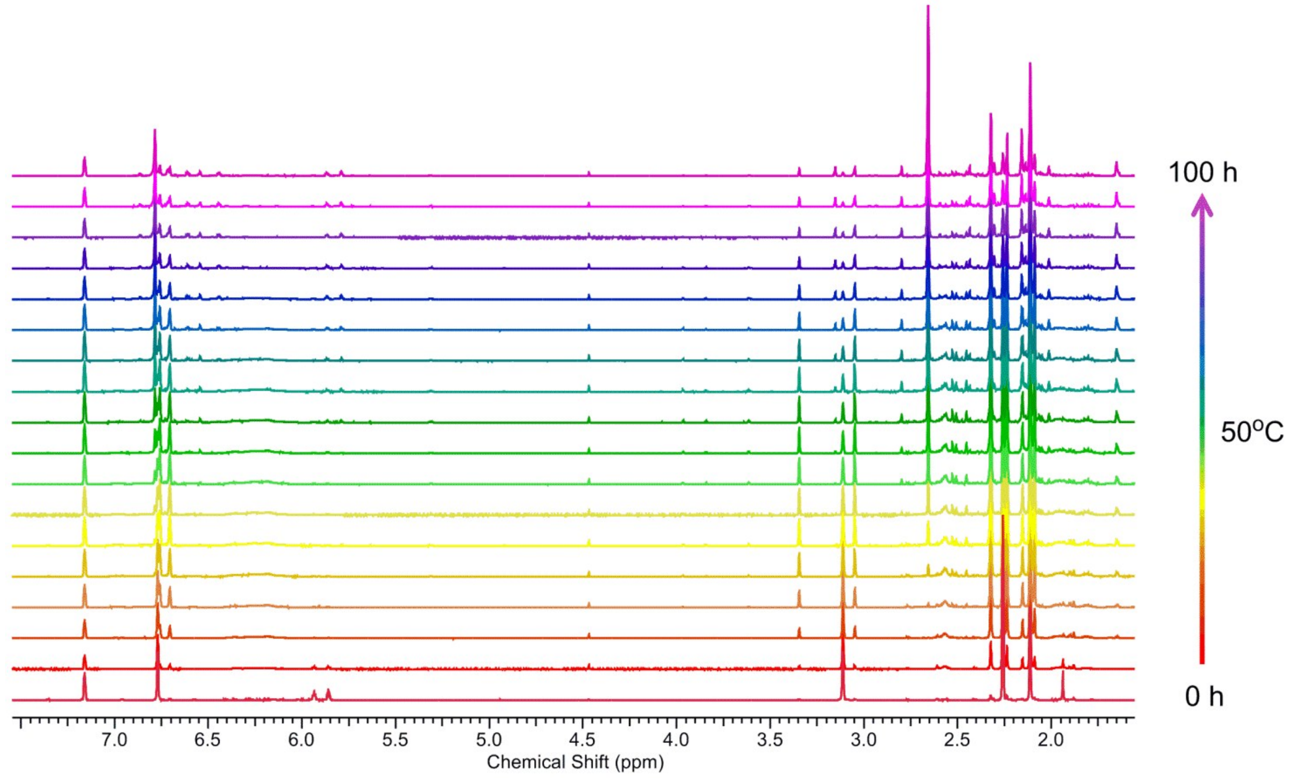

Figure S19. Variable-time ^{1}H NMR spectrum of the dehydrocoupling of $MesSbH_2$ catalysed by 10 mol% Cp'_3Y. Temperature = 50°C.

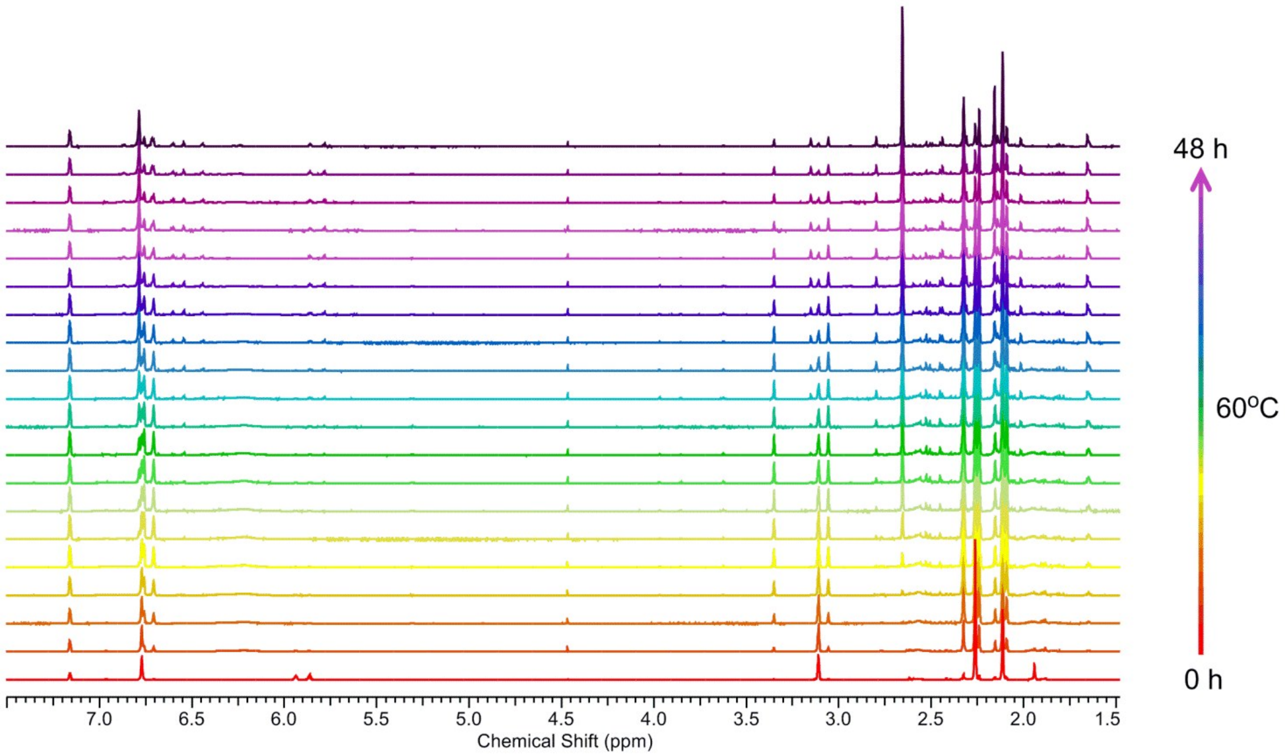

Figure S20. Variable-time ^{1}H NMR spectrum of the dehydrocoupling of $MesSbH_2$ catalysed by 10 mol% Cp'_3Y. Temperature = 60°C.

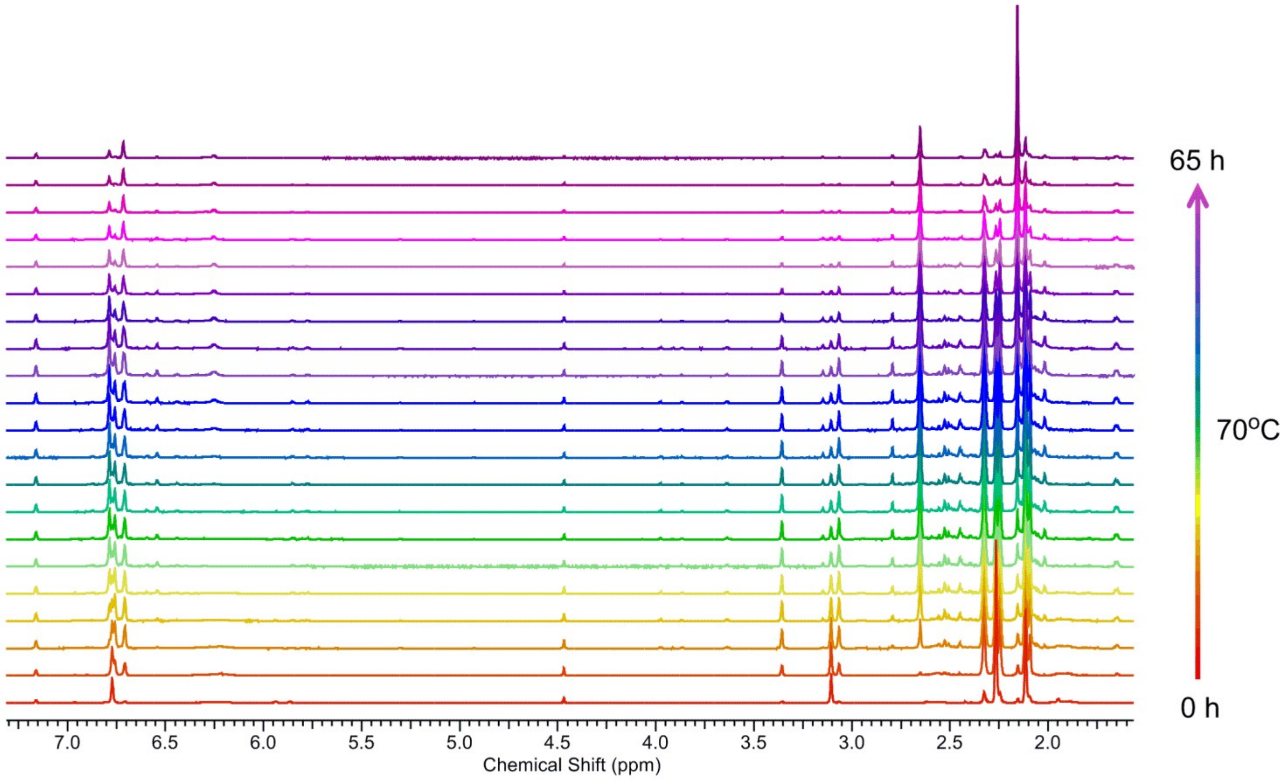

Figure S21. Variable-time ^{1}H NMR spectrum of the dehydrocoupling of $MesSbH_2$ catalysed by 10 mol% Cp'_3Y. Temperature = 70°C.

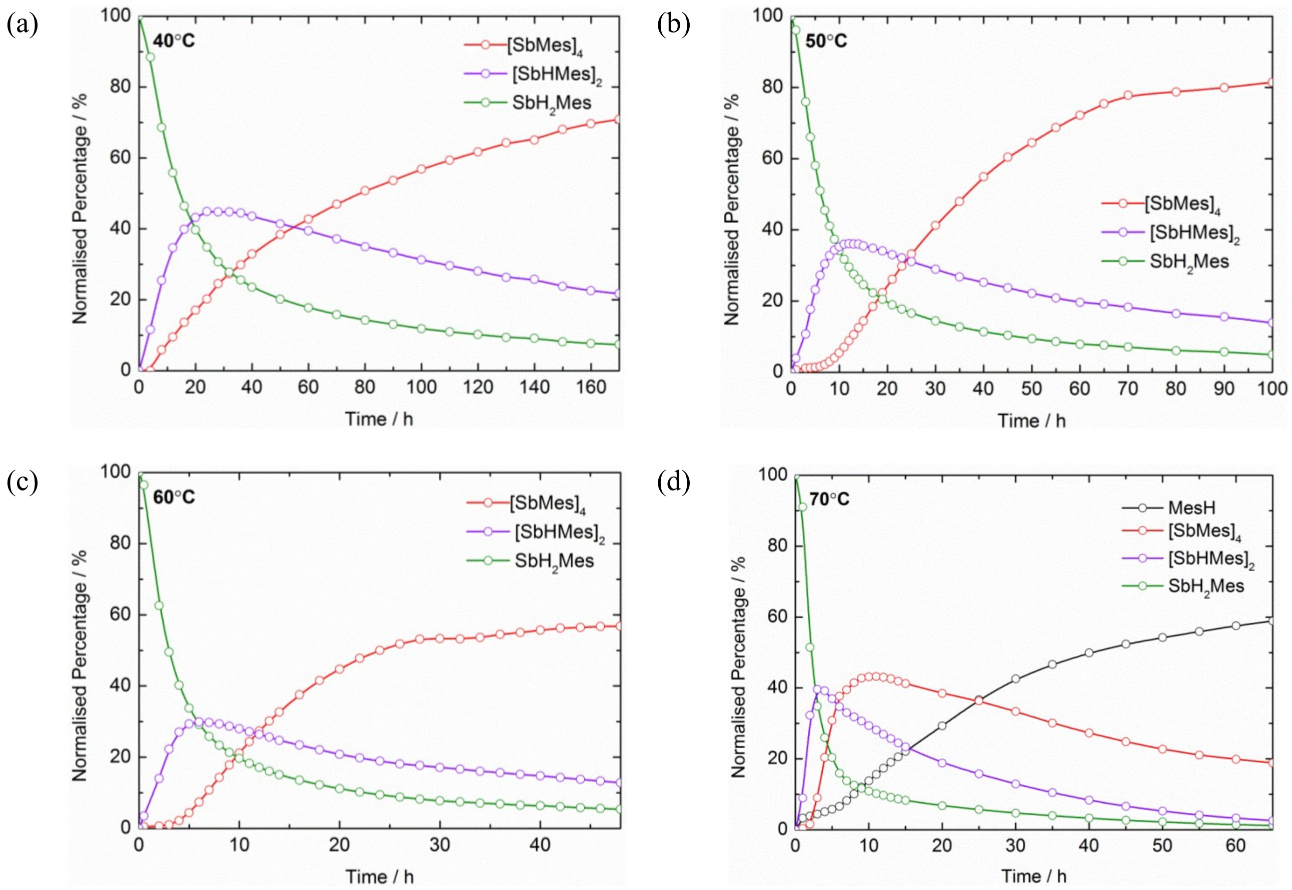

Figure S22. Product distributions from the dehydrocoupling of $MesSbH_2$ catalysed by 10 mol% Cp'_3Y at: (a) 40°C; (b) 50°C; (c) 60°C; (d) 70°C. Relative amounts of each component were determined by integration of distinct resonances in the ^1H NMR spectra shown in Figures S18-S21.

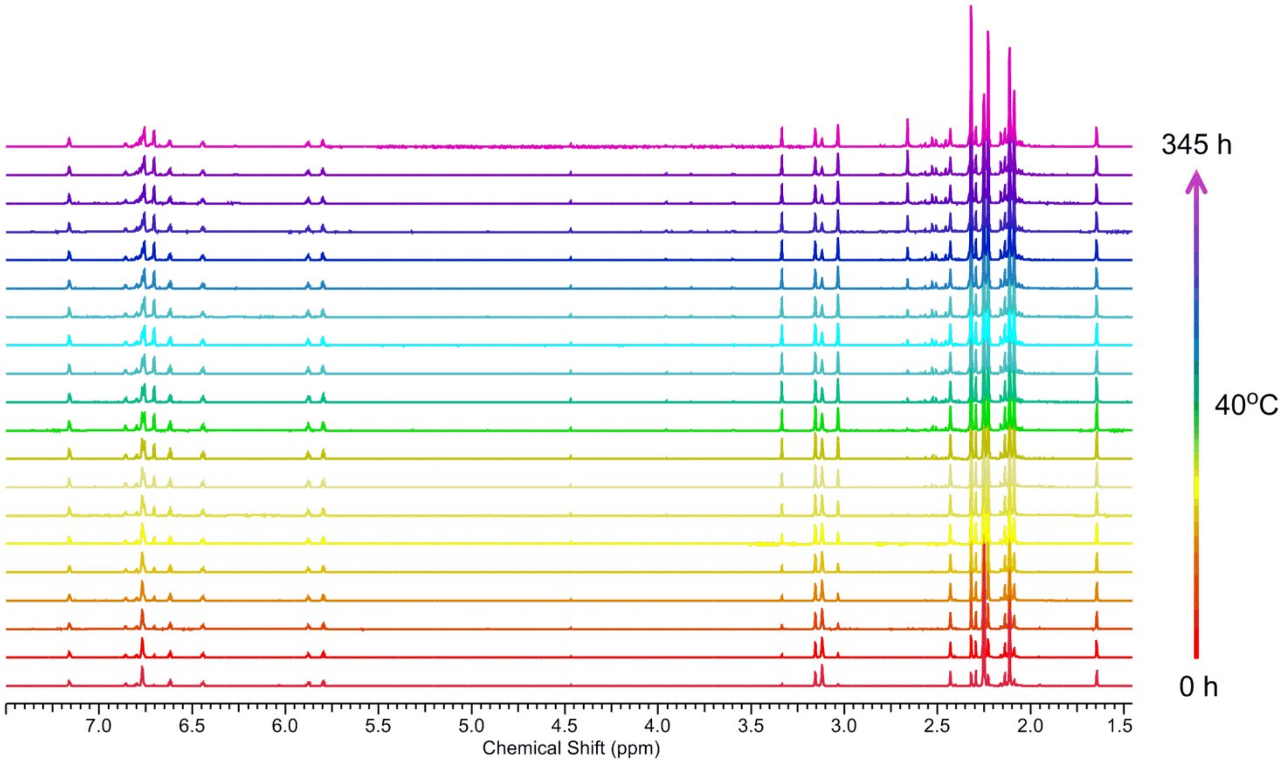

Figure S23. Variable-time ^1H NMR spectrum of the dehydrocoupling of $MesSbH_2$ catalysed by 3.33 mol% **2-Y**. Temperature = 40°C.

References

1. Breunig, H. J., Ghesner, M. E., Lork, E. Syntheses of the antimonides R_2Sb^- R = Ph, Mes, *t*Bu, *t*Bu_2Sb) and Sb_7^{3-} by reaction of organoantimony hydrides or *cyclo*-(*t*BuSb)$_4$ with Li, Na, K or BuLi. *Z. Anorg. Allg. Chem*. **631**, 851-856 (2005).
2. Wilkinson, G. & Birmingham, J. M. Cyclopentadienyl compounds of Sc, Y, La, Ce and some lanthanide elements. *J. Am. Chem. Soc*. **76**, 6210-6210 (1954).
3. Aquilante, F. *et al*. Molcas 8: New capabilities for multiconfigurational quantum chemical calculations across the periodic table. *J. Comput. Chem.* **37**, 506-41 (2016).
4. Neese, F. The ORCA program system. *WIREs Comput. Mol. Sci*. **2**, 73-78 (2012).
5. Perdew, J. Density-functional approximation for the correlation energy of the inhomogeneous electron gas. *Phys. Rev. B* **33**, 8822-8824 (1986).
6. Becke, A. D. Density-functional exchange-energy approximation with correct asymptotic behavior. *Phys. Rev. A* **38**, 3098-3100 (1988).
7. Schäfer, A., Horn, H., Ahlrichs, R. Fully optimized contracted Gaussian basis sets for atoms Li to Kr. J. Chem. Phys. 97, 2571-2577 (1992).
8. Weigend, F., Ahlrichs, R. Balanced basis sets of split valence, triple zeta valence and quadruple zeta valence quality for H to Rn: Design and assessment of accuracy. *Phys. Chem. Chem. Phys*. **7**, 3297-3305 (2005).
9. Grimme, S., Ehrlich, S., Goerigk, L. Effect of the damping function in dispersion corrected density functional theory. *J. Chem.* **32**, 1456-1465 (2011)
10. Roos, B. O., Lindh, R., Malmqvist, P.-Å., Veryazov, V., Widmark, P.-O. New relativistic ANO basis sets for transition metal atoms. *J. Phys. Chem. A* **109**, 6575-6579 (2005).
